# The Use of Intravenous Clonidine in Postoperative Pain Management: A Scoping Review

**DOI:** 10.1111/aas.70329

**Published:** 2026-08-27

**Authors:** Tórálvur Mohr, Emma Atsuko Tsuchiya, Mette Krag, Bitten Dybdal, Johan Heiberg

**Affiliations:** ^1^ Department of Anaesthesiology, Surgery and Trauma Centre Copenhagen University Hospital – Rigshospitalet Copenhagen Denmark; ^2^ Department of Clinical Medicine University of Copenhagen Copenhagen Denmark

## Abstract

**Background:**

Clonidine, an alpha‐2 receptor agonist, has been explored as a potential analgesic in postoperative pain management, but its efficacy and safety profile remain unclear. The aim of this scoping review was to present the evidence on the use of intravenous clonidine in postoperative pain management.

**Methods:**

This scoping review was conducted following the Preferred Reporting Items for Systematic Reviews and Meta‐Analyses Extension for Scoping Reviews. We searched the following databases: The Cochrane Library, Embase, and MEDLINE for studies investigating the use of intravenous clonidine in postoperative pain management, regardless of comparators or outcomes. Data were summarized and presented descriptively. Risk of bias was assessed using the Risk of Bias 2 tool for randomized trials, while the certainty of evidence was evaluated at the outcome level using a modified Grading of Recommendations Assessment, Development and Evaluation approach.

**Results:**

We included 10 trials with a total of 646 patients. Most patients were ASA I–II and underwent major elective surgery. The trials varied in clonidine dosage, timing, and combination with other analgesics. All included trials reported outcome data for pain and/or supplemental analgesia with high variability in assessment strategies. The data showed inconsistent effects of clonidine in reducing pain scores and the need for supplemental analgesia. Reported adverse effects were also inconsistent across studies. The overall certainty of evidence was very low for all outcomes.

**Conclusion:**

Evidence for the use of intravenous clonidine in postoperative pain management is heterogeneous and limited, providing no consistent clinical benefit and underscoring the need for further research.

**Editorial Comment:**

This scoping review presents the current evidence for intravenous clonidine for post‐operative analgesia. So far there is not enough evidence to support a recommendation concerning if or how intravenous clonidine should be used in this context.

## 1 Introduction

Postoperative pain management is an essential part of patient care. Effectively managing patients' pain can promote recovery, improve outcomes, and increase patient satisfaction [1]. For years, opioids have been a cornerstone of postoperative pain management. However, evidence supports the use of multimodal analgesia, integrating various classes of pain‐relieving agents [2]. These strategies aim to reduce opioid use, thereby diminishing the risk of tolerance and escalation while achieving equal or superior pain relief.

One potential non‐opioid analgesic is clonidine, an alpha‐2 receptor agonist. Clonidine was originally developed to treat hypertension [3], but has been shown to exert analgesic effects via spinal and supraspinal mechanisms [4, 5]. Clonidine has also been shown to increase the efficacy of various analgesics, such as opioids [6, 7] and local analgesics [8, 9, 10]. This is, however, an off‐label indication and the evidence on the effects and safety of intravenous clonidine is unclear.

With this scoping review, we aimed to systematically outline the evidence on intravenously administered clonidine for postoperative pain management in adult patients to identify unanswered research questions. We hypothesized that the evidence is limited and derived from heterogeneous populations.

## 2 Methods

This scoping review was conducted in accordance with the Preferred Reporting Items for Systematic Reviews and Meta‐Analyses Extension for Scoping Reviews [11]. The completed PRISMA‐ScR checklist can be found in the Supporting Information (Table S1). We addressed the following research questions as follows:

− For which patients and procedures has postoperative administration of clonidine been assessed?
− Which doses and timings of clonidine administration have been studied for postoperative pain management?
− Which combinations of analgesics and postoperatively administered clonidine have been evaluated in the context of postoperative pain management?
− Which desirable and undesirable outcomes of intravenous clonidine have been assessed in postoperative pain management?

### 2.1 Eligibility Criteria

We included all studies assessing the use of postoperatively administered intravenous clonidine for postoperative pain management in adult patients. The comparator of interest was any other intravenously administered drug for postoperative pain management or placebo. We included all study designs except review articles and case reports. Identified conference abstracts that were not published as full articles were excluded. We excluded studies on animals and children, and studies in languages other than English. There were no restrictions on comparators, reported outcomes, publication year, or blinding.

### 2.2 Information Sources and Search Strategy

We systematically searched the following databases: The Cochrane Library, Embase, and MEDLINE. The electronic search strategy, provided in the Supporting Information: Appendix 1, was developed in collaboration with an information specialist and was tested and revised before the final literature search on November 25, 2025.

### 2.3 Study Selection

Study selection was performed using Covidence software. Two review authors (T.M. and E.A.T.) independently screened titles and abstracts and excluded studies that were clearly irrelevant. Thereafter, the full texts of the included studies were assessed for eligibility. Any discrepancies were resolved through discussion between the screening authors or, if necessary, by arbitration with a third author (J.H., M.K.).

### 2.4 Data Extraction and Synthesis

One author (T.M.) extracted the characteristics of all included studies using a predefined data extraction sheet, which can be found in the Supporting Information (Table S2). The extracted data included publication year, country, study design, surgical procedure, setting, intervention and comparator, and reported outcomes. Data were summarized and presented descriptively.

### 2.5 Outcome Measures

All reported outcome measures were included, encompassing pain assessment, supplemental analgesia, and adverse effects.

### 2.6 Risk of Bias Assessment

One author (T.M.) assessed the risk of bias for the included trials using the Cochrane Risk of Bias 2 tool (RoB 2) [12]. The risk of bias assessment was conducted separately for each outcome measure across the five RoB 2 domains: bias arising from the randomization process, bias due to deviations from the intended interventions, bias due to missing outcome data, bias in measurement of the outcome, and bias in selection of the reported result. Each domain was judged to have low risk of bias, some concerns, or high risk of bias. Uncertainties in the assessment were discussed with a second author (E.T.).

### 2.7 Assessment of the Certainty of Evidence

The certainty of evidence on outcome level was assessed using a modified approach of the Grading of Recommendations Assessment, Development, and Evaluation (GRADE) tool [13, 14]. Using this framework, the certainty of evidence for each outcome was rated as high, moderate, low, or very low. The assessment considered the GRADE domains of risk of bias, inconsistency, indirectness, imprecision, and publication bias, and evidence was downgraded or upgraded according to GRADE criteria. Two authors (T.M. and E.A.T.) independently assessed the certainty of evidence for each outcome. GRADEpro GDT software was used to conduct the GRADE assessments and to create the Summary of Findings table. We used the guidance described by Santesso et al. [15] to communicate our findings.

## 3 Results

**FIGURE 1:**
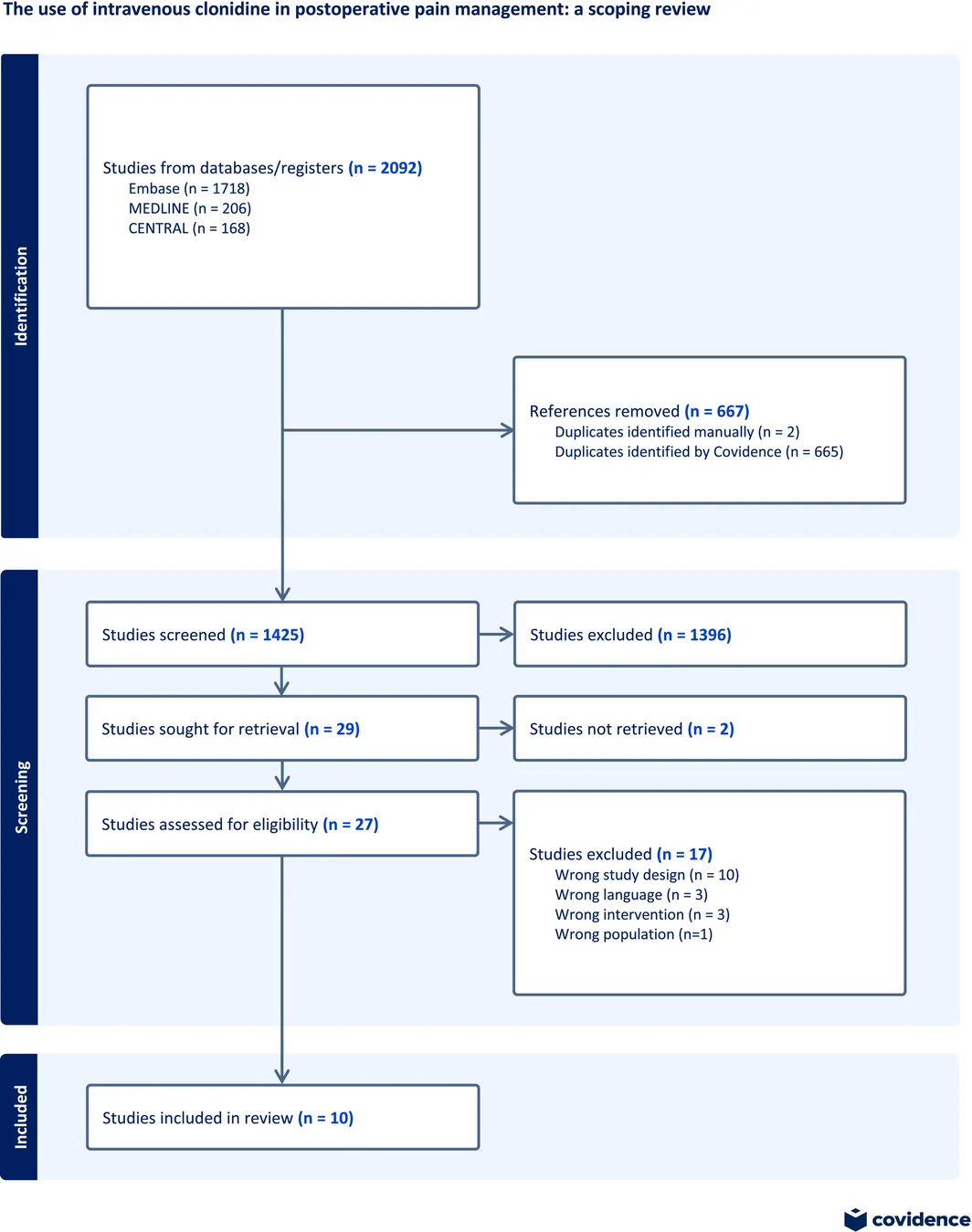
PRISMA flow diagram: Identification of studies assessing intravenous clonidine in postoperative pain management.

The primary search identified 1577 studies, of which 10 were included in this review. These studies were conducted in seven different countries and included a total of 646 patients. All included studies were randomized clinical trials published between 1991 and 2024. The study selection process is illustrated in the PRISMA flow diagram (Figure 1).

### 3.1 Characteristics of Studies

#### 3.1.1 Populations

All trials included adult patients undergoing elective surgery, of whom 71% were classified as American Society of Anesthesiologists (ASA) Grade I–II. The surgical procedures were primarily abdominal (59%) and orthopedic (30%).

#### 3.1.2 Interventions and Comparators

Five trials (378 patients) assessed clonidine as monotherapy compared with placebo [7, 16, 17, 18, 19]. One of these trials also included a dexmedetomidine group as a second comparator [16]. In five trials, the intervention was clonidine in combination with an opioid [20, 21, 22, 23, 24], and in two of these trials this intervention was also combined with ketamine [20, 24]. In three of the trials the comparator consisted of the same analgesic regimens as the intervention group, but without clonidine [21, 22, 23]; one used placebo [24], and one compared administration of a morphine‐ketamine‐clonidine combination at the start of surgery with administration at extubation [20]. Further trial characteristics are presented in Table 1.

**TABLE 1:** Characteristics of studies on postoperative pain management comparing intravenous clonidine with any other intervention.

Author, year, country	Study design	Population	Setting	Intervention	Comparator	Outcomes	Pain assessment strategy
Bedi [16], 2023, India	RCT, double blind	Elective laparotomies 105 ASA I‐III	10 min prior to anticipated end of surgery	$3\mathrm{\mu g}\cdot {\mathrm{kg}}^{-1}$ of clonidine diluted up to 10 mL normal saline given over 10 min i.v.	$1\mathrm{\mu g}\cdot {\mathrm{kg}}^{-1}$ of dexmedetomidine diluted up to 10 mL normal saline given over 10 min i.v. OR Placebo	Pain Sedation Hemodynamics Other	VAS 0‐10 cm Every 5 min until 30 min and then every 15 for the next 30 min
Bernard [7], 1991, France	RCT, single blinded	Elective spinal surgery 50 patients ASA I	At admission to the recovery room	I.v. clonidine $5\mathrm{\mu g}\cdot {\mathrm{kg}}^{-1}$ infused over the first hour Thereafter a maintenance dose of $0.3\mathrm{\mu g}\cdot {\mathrm{kg}}^{-1}\cdot h^{-1}$ for 11 h.	Placebo	Pain Supplemental analgesia Sedation Hemodynamics Respiration Other	VAS 0‐100 mm Before administration, after 1 h and thereafter every 2 h for 10 h.
Bernard [23], 1994, France	RCT, double blinded	Elective scoliosis correction 30 patients ASA I	At extubation in the operating room	I.v. fentanyl‐clonidine combination of $25\mathrm{\mu g}\cdot h^{-1}$and $0.3\mathrm{\mu g}\cdot {\mathrm{kg}}^{-1}\cdot h^{-1}$, respectively, continued for 14–16 h	I.v. fentanyl of $75\mathrm{\mu g}\cdot h^{-1}$ continued for 14–16 h	Pain Supplemental analgesia Sedation Hemodynamics Respiration Other	Pain: VAS 0‐100 mm measurements before and 15, 30, and 120 min after the loading dose started, and then every 2 h for 8 h.
De Kock [21], 1994, Belgium	RCT, single blinded	Elective left colonic resection for neoplastic disease 80 patients ASA I‐II	At skin incision in operating room Thereafter administrated via PCA	I.v. clonidine $4\mathrm{\mu g}\cdot {\mathrm{kg}}^{-1}$ over 20 min followed by $2\mathrm{\mu g}\cdot {\mathrm{kg}}^{-1}\cdot h^{-1}$ until closure of peritoneum Thereafter patient administered i.v. clonidine 15μg and morphine 1.5mg with lockout intervals of 7 min	Placebo Thereafter patient administered i.v. morphine 2mg with lockout intervals of 7 min or I.v. clonidine $4\mathrm{\mu g}\cdot {\mathrm{kg}}^{-1}$ over 20 min followed by $2\mathrm{\mu g}\cdot {\mathrm{kg}}^{-1}\cdot h^{-1}$ until closure of peritoneum Thereafter patient administered i.v. morphine 1.5mg with lockout intervals of 7 min	Pain Supplemental analgesia Sedation Hemodynamics Respiration Other	Pain: Four‐point analgesia scale: 0 = no pain; 1 = moderate, good control with PCA; 2 = moderate pain, poor control with PCA; 3 = unbearable pain. Pain measurements were determined at 6, 12, 18, 24, and 36 postoperative hours. Also, patients evaluated their overall postoperative pain relief at the end of observation by a 3‐point scale.
Holthusen [20], 2002, Germany	Randomized prospective trial, double blinded	Elective transperitoneal tumor nephrectomy 30 patients ASA I‐III	Intervention group: At extubation in operating room Control group: At skin incision in operating room	I.v clonidine $5\mathrm{\mu g}\cdot {\mathrm{kg}}^{-1}$, morphine $150\mathrm{\mu g}\cdot {\mathrm{kg}}^{-1}$, and ketamine $150\mathrm{\mu g}\cdot {\mathrm{kg}}^{-1}$ over 15 min	I.v clonidine $5\mathrm{\mu g}\cdot {\mathrm{kg}}^{-1}$, morphine $150\mathrm{\mu g}\cdot {\mathrm{kg}}^{-1}$, and ketamine $150\mathrm{\mu g}\cdot {\mathrm{kg}}^{-1}$ over 15 min	Pain Supplemental analgesia Other	Pain: VAS 0–10 at rest and during induced cough: Assessed every 2 for the first 8 h, then every 8 for 48 h. Assessed at rest and at induced cough.
Jeffs [22], 2002, United Kingdom	RCT, double blinded	Elective, major lower abdominal or gynecological surgery 60 patients ASA I‐II	At abdominal closure in operating room Thereafter administrated via PCA	I.v. clonidine $4\mathrm{\mu g}\cdot {\mathrm{kg}}^{-1}$ over 20 min Thereafter patient administrated doses of i.v. clonidine 20μg and morphine 1mg clonidine with lockout intervals of 5 min	Placebo Thereafter patient administrated doses of i.v morphine 1mg with lockout intervals of 5 min	Pain Supplemental analgesia Sedation Other	Pain: Four‐point score: 0 = no pain; 1 = mild pain; 2 = moderate pain; 3 = severe pain Pain was assessed 12, 24, and 36 h after surgery. Postoperative ain relief satisfaction was also assessed 36 h after surgery.
Liu [17], 2024, Australia	RCT, double blinded	Elective, non‐cardiac surgery, 83 patients APACHE II 8–12	At arrival to HDU	$0.3\mathrm{\mu g}\cdot {\mathrm{kg}}^{-1}\cdot h^{-1}$ clonidine by 5 mL/h i.v. infusion until 6 AM on first postoperative day	Placebo	Supplemental analgesia Hemodynamics Other	Not reported
Marinangeli [18], 2002, Italy	RCT, double blinded	Elective, lumbar hemilaminectomy 80 patients ASA I‐II	Approximately 30 min before end of surgery Thereafter continuous infusion	Loading dose of i.v. clonidine: Group A: $5\mathrm{\mu g}\cdot {\mathrm{kg}}^{-1}$ Group B: $3\mathrm{\mu g}\cdot {\mathrm{kg}}^{-1}$ Group C: $2\mathrm{\mu g}\cdot {\mathrm{kg}}^{-1}$ Thereafter equal infusion of i.v. clonidine $0.3\mathrm{\mu g}\cdot {\mathrm{kg}}^{-1}\cdot h^{-1}$ for 12 h	Placebo	Pain Supplemental analgesia Sedation Hemodynamics Other	Met/unmet PCA requests and VAS at rest after 1, 2, 3, 4, 5, 6, 9, 12 h.
Salengros [24], 2011, Belgium	RCT, double blinded	Elective, orthopedic, abdominal, gynecological, vascular, and head and neck surgery 68 patients ASA I‐III	At first complaint of pain in recovery room	If NRS ≥ 5 then standard opioid‐based analgesic protocol Thereafter, IF NRS ≥ 4: I.v. clonidine‐ketamin combination. $0.5\mathrm{\mu g}\cdot {\mathrm{kg}}^{-1}$ and $0.125\mathrm{mg}\cdot {\mathrm{kg}}^{-1}$, respectively, over 15 min	If NRS ≥ 5 then Standard opioid‐based analgesic protocol Thereafter, IF NRS ≥ 4: Placebo	Pain Supplemental analgesia Sedation Hemodynamics Respiration Other	11‐point NRS at PACU arrival. Thereafter measured at the end of study drug infusion. Time to NRS < 4
Striebel [19], 1993, Germany	RCT, double blinded	Elective, cholecystectomy 60 female patients ASA I‐II	At first complaint of pain in recovery room	I.v. clonidine 150μg over 30 min Thereafter i.v. clonidine 150μg over the following 90 min	Variable loading doses	Pain Supplemental analgesia Hemodynamics Respiration Other	Pain: VAS 0‐100 mm, which was assessed right before infusion of clonidine or placebo and every 10 min thereafter for a period of 2 h.

Abbreviations: APACHE, Acute Physiology and Chronic Health Evaluation; ASA, American Society of Anesthesiology; CA, Patient‐Controlled Analgesia; h, hours; HDU, High‐Dependency Unit; i.v., intravenous; min, minutes; NRS, numeric rating scale; PACU, Post‐Anaesthesia Care Unit; RCT, Randomized Controlled Trial.

### 3.2 Outcomes

The included trials reported various outcome parameters. Nine trials (563 patients) reported on pain scores [7, 16, 18, 19, 20, 21, 22, 23, 24] using different assessment methods. Six trials used a Visual Analog Scale [7, 16, 18, 19, 20, 23], two used a four‐point pain score [21, 22], and one used a Numeric Rating Scale [24]. Nine trials (541 patients) reported on supplemental analgesia [7, 17, 18, 19, 20, 21, 22, 23, 24]; eight trials (511 patients) reported on the use of opioids [7, 17, 18, 19, 20, 21, 22, 24], and one trial (30 patients) reported on the use of a nonsteroidal anti‐inflammatory drug (ketoprofen) [23].

#### 3.2.1 Risk of Bias

**FIGURE 2:**
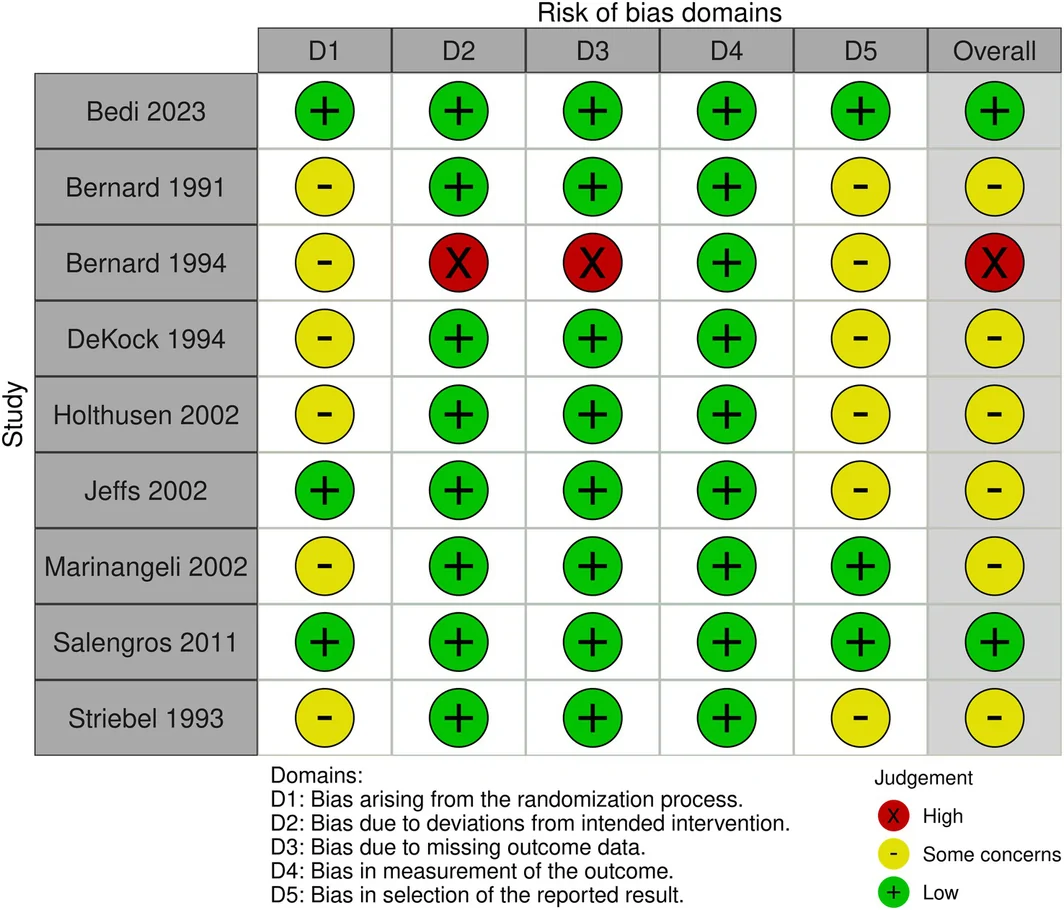
Risk of bias assessment for the outcome of pain scores using Cochrane's RoB2‐tool.

**FIGURE 3:**
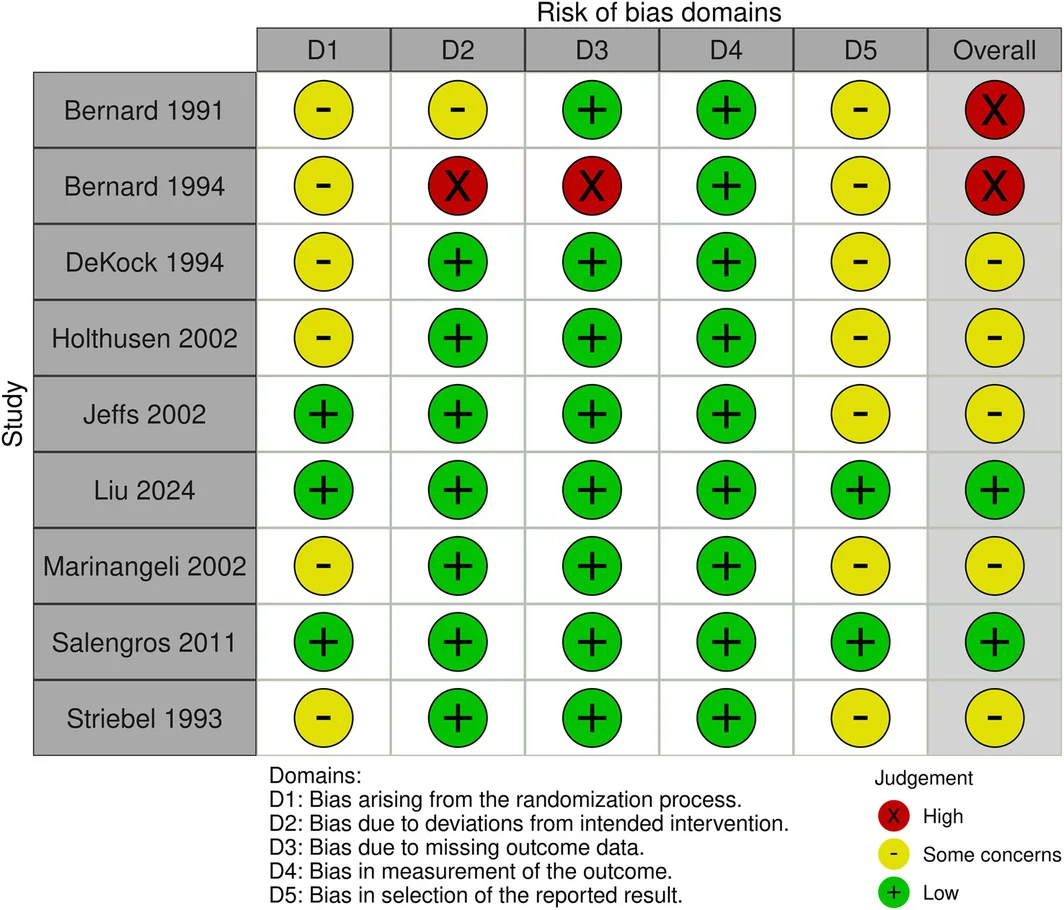
Risk of bias assessment for the outcome of supplemental analgesia using Cochrane's RoB2‐tool.

**FIGURE 4:**
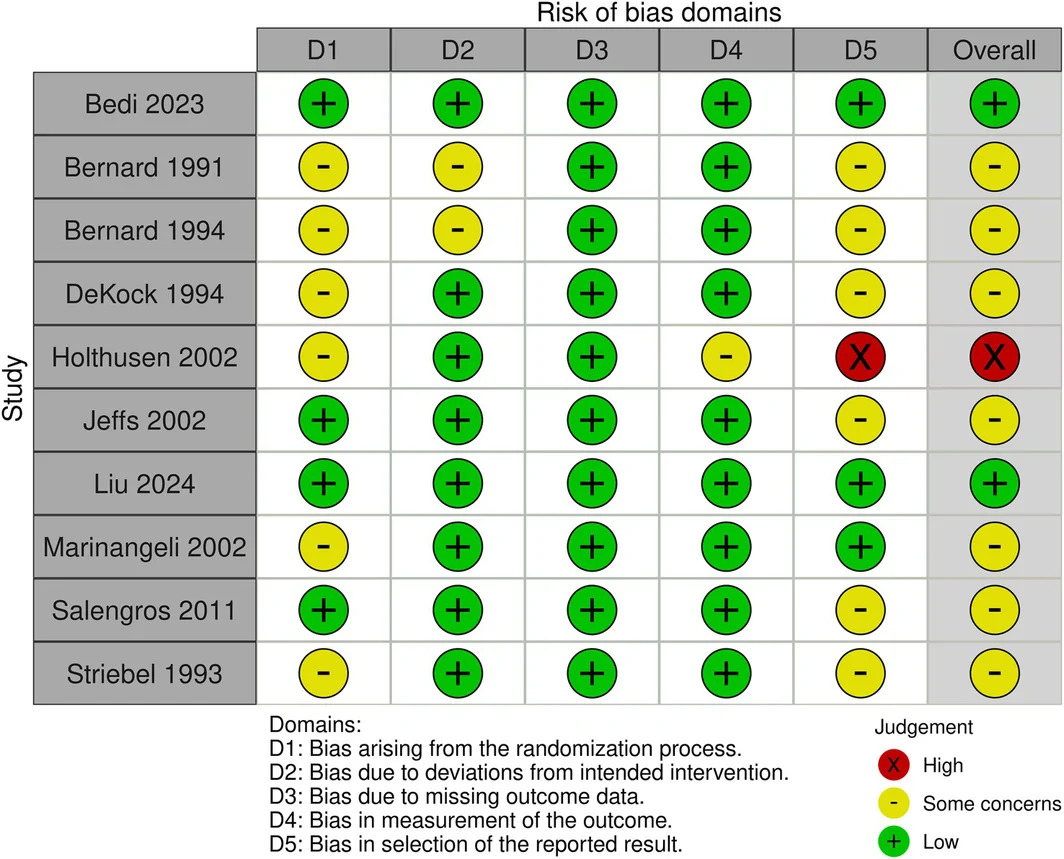
Risk of bias assessment for the outcome of adverse effects using Cochrane's RoB2‐tool.

Across all outcome measures, most of the included trials were judged to have some concerns regarding risk of bias. This was most frequently due to bias arising from the randomization process and bias in the selection of the reported result. The risk of bias assessments for each outcome measure is presented in Figures 2, 3, and 4. Detailed domain‐level assessments are provided in the Supporting Information (Table S3).

#### 3.2.2 Pain and Supplemental Analgesia

In the five trials comparing clonidine as monotherapy with placebo, two trials (130 patients) reported significant reductions in both postoperative pain scores and supplemental analgesia consumption [7, 18]. One trial (105 patients) reported a significant reduction in pain scores but did not report supplemental analgesia [16]. One trial (60 patients) found no differences in pain scores [19], while two trials (143 patients) found no differences in supplemental analgesia [17, 19].

In trials where the intervention was clonidine in combination with other analgesics, one trial found reduced pain by adding clonidine to morphine compared with morphine alone [22], and one trial found lower pain scores and reduced morphine requirements when perioperative clonidine followed by patient‐controlled clonidine and morphine was compared with placebo followed by patient‐controlled morphine, but not when compared with perioperative clonidine followed by patient‐controlled morphine [21]. The remaining trials found no statistically significant differences in either pain scores or supplemental analgesia [20, 23, 24]. One of those trials found similar reductions in pain scores and supplemental analgesia when comparing clonidine with 25 μg·h^−1^ fentanyl versus 75 μg·h^−1^ fentanyl with no clonidine [23], and another found increased postoperative pain‐relief satisfaction when clonidine was added to morphine compared with morphine alone [22].

The certainty of evidence for pain scores and supplemental analgesia was very low, downgraded for risk of bias, indirectness, and imprecision (Table 2).

**TABLE 2:** GRADE quality of evidence assessment.

Outcome	No. of studies	Study designs	Risk of bias	Inconsistency	Indirectness	Imprecision	Publication bias	Quality of evidence^d^
Pain scores	9	RCTs	Serious^a^	Not serious	Serious^b^	Serious^c^	Not detected	Very low
Supplemental analgesia	9	RCTs	Serious^a^	Not serious	Serious^b^	Serious^c^	Not detected	Very low
Adverse effects	10	RCTs	Serious^a^	Not serious	Serious^b^	Very serious^c^	Not detected	Very low

^a^ see RoB2 figures for in‐depth risk of bias assessments.

^b^ there was substantial variation across studies in interventions and assessment methods, limiting the applicability of the evidence to a single clinical scenario.

^c^ small sample sizes and limited overall statistical power across trials.

^d^ Grading of Recommendations, Assessments, Development and Evaluation (GRADE).

Reported outcomes on pain and supplemental analgesia for all trials can be seen in Table 3.

**TABLE 3:** Pain and supplemental analgesia outcomes from studies comparing postoperative pain management with intravenous clonidine with placebo or other intervention.

Studies giving clonidine as monotherapy				
Study	Comparator	Pain outcome	Supplemental analgesia outcome	Summary
Bedi [16], 2023	Placebo or Dexmedetomidine	Significant or Not significant	Not reported	Clonidine reduced pain scores versus placebo between 20 and 60 min post‐extubation (median VAS 1 point lower in the clonidine group). Pain scores did not differ significantly from dexmedetomidine. Supplemental analgesia was not reported.
Bernard [7], 1991	Placebo	Significant	Significant	Clonidine reduced VAS pain scores versus placebo from the end of the loading dose and at 2‐h assessments over the subsequent 10 h (VAS 26 ± 3 mm vs. 42 ± 5 mm) at the end of loading dose. Total morphine requirement was lower in the clonidine group (3.8 ± 1 mg vs. 10.8 ± 1.2 mg).
Liu [17], 2024	Placebo	Not reported	Not significant	Clonidine did not reduce postoperative opioid consumption versus placebo
Marinangeli [18], 2002	Different loading doses, followed by infusion	Significant	Significant	Clonidine reduced VAS scores and morphine requirement in a dose‐dependent manner over 12 h. Morphine doses requested: 5 ± 2 ($5\mathrm{\mu g}\cdot {\mathrm{kg}}^{-1}$), 11 ± 3 ($3\mathrm{\mu g}\cdot {\mathrm{kg}}^{-1}$), 19 ± 4 ($2\mathrm{\mu g}\cdot {\mathrm{kg}}^{-1}$), and 29 ± 8 (placebo). VAS scores were reported graphically; numerical estimates not provided. The $2\mathrm{\mu g}\cdot {\mathrm{kg}}^{-1}$ group did not differ from placebo on VAS.
Striebel [19], 1993	Placebo	Not significant	Not significant	Clonidine did not reduce pain and supplemental analgesia versus placebo
Studies giving clonidine in combination with other analgesics				
Bernard [23], 1994	Fentanyl	Not significant	Not significant	Clonidine combined with $25\;\mathrm{\mu g}\cdot {\mathrm{kg}}^{-1}$ fentanyl did not decrease pain or supplemental analgesia versus $75\;\mathrm{\mu g}\cdot {\mathrm{kg}}^{-1}$ fentanyl
DeKock [21], 1994	Intraoperative placebo thereafter PCA morphine or Intraoperative clonidine thereafter PCA morphine	Significant or Not significant	Significant or Not significant	The intervention group, receiving intraoperative clonidine and postoperative PCA clonidine with morphine, had lower pain scores and reduced morphine requirements compared with the group receiving postoperative PCA morphine alone at all‐time points assessed (6–36 h).
Holthusen [20], 2002	Administration at start of surgery	Not significant	Not significant	There was no difference in pain and supplemental analgesia between administering clonidine, morphine and ketamine at start of surgery versus end of surgery
Jeffs [22], 2002	Placebo thereafter PCA morphine	Transiently significant	Transiently significant	Clonidine combined with morphine reduced pain compared to morphine alone at 12 h (median verbal pain score on a 4‐point scale: 1 vs. 3 at 12 h), with no significant difference thereafter. Mean cumulative morphine consumption was lower in the clonidine group at 36 h (15 mg vs. 21 mg), with no significant differences at 12 or 24 h.
Salengros [24], 2011	Placebo	Not significant	Not significant	There was no difference in pain and supplemental analgesia when giving clonidine combined with ketamine versus placebo

Abbreviations: min, minutes; PCA, patient controlled analgesia; VAS, Visual Analog Scale; vs., versus.

### 3.3 Adverse Effects

**FIGURE 5:**
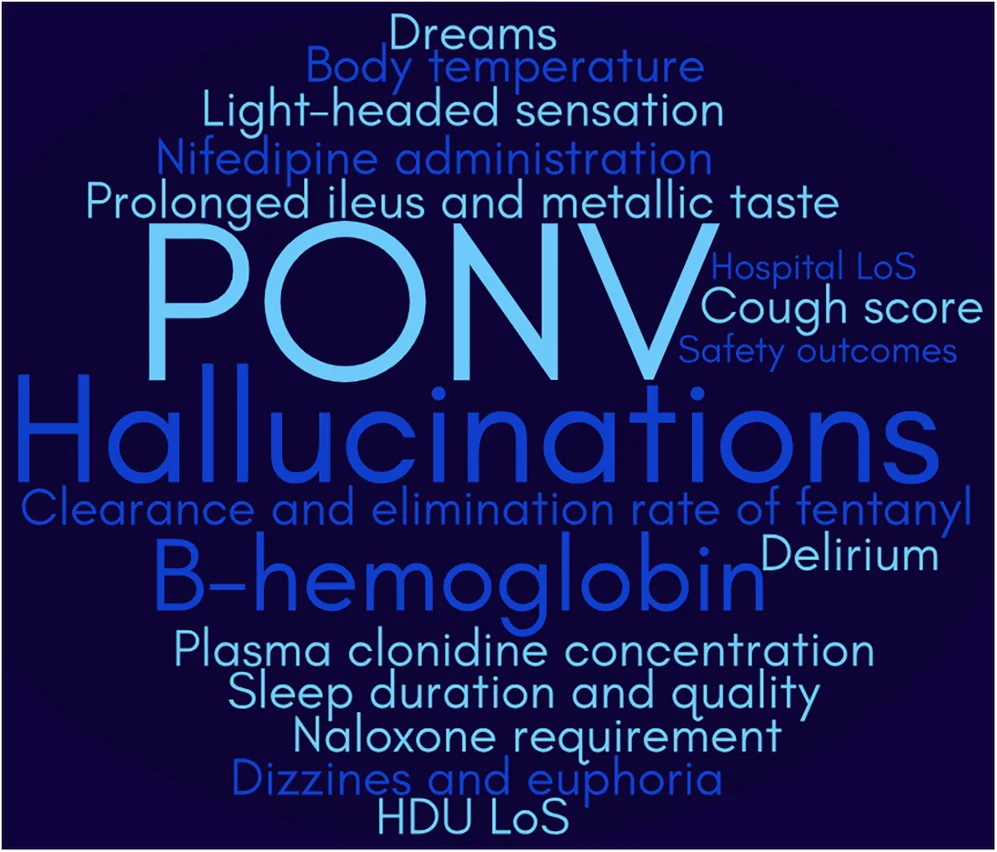
Word cloud of adverse effects grouped under the ‘other’ category. The size of each term reflects reporting frequency across studies, with PONV being the most reported outcome (*n* = 5).

All trials reported on adverse effects (Table 4 and Figure 5). Sedative effects were the only neurological adverse effects reported and were reported in seven trials (473 patients) using different assessment scales [7, 16, 18, 21, 22, 23, 24]. Two trials (185 patients) reported greater sedation in the clonidine group compared with placebo [16, 18]. Four trials (208 patients) found no statistically significant differences between groups [7, 22, 23, 24].

**TABLE 4:** Adverse effects from studies comparing postoperative pain management with intravenous administration of clonidine with placebo or other intervention.

Outcome category	Specific outcome measures	Total number of studies	Summary
Sedation	Sedation	Seven studies [7, 16, 18, 21, 22, 23, 24]	One study reported a decreased sedation level in the group receiving intraoperative and postoperative i.v. clonidine compared to morphine, but no significant difference comparing a group receiving only intraoperative clonidine [21]. Two studies found an increase in sedation in the groups receiving i.v. clonidine compared to placebo [20, 21, 22]. Four studies found no significant difference in sedation levels between groups [7, 22, 23, 24]
Hemodynamics	Heart rate	Six studies [7, 16, 17, 18, 19, 23]	Three studies found a decrease in heart rate [7, 18, 19]. Three studies found no significant difference in heart rate [16, 17, 23].
	Blood pressure	Seven studies [7, 16, 17, 18, 19, 23, 24]	Two studies found a decrease in mean arterial pressure [7, 23], two studies found a decrease in systolic blood pressure [18, 19]. Three studies found no significant difference in hypotension between groups [16, 17, 24].
	Other cardiac parameters	Three studies [7, 21, 23]	One study found decreased cardiac index and systemic vascular resistance index [7]. One study found no significant difference in right atrial pressure, mean pulmonary artery pressure or pulmonary capillary wedge pressure [7], and one study found no significant difference in mean pulmonary artery pressure and cardiac index between groups [23]. One study found a decrease in hemodynamic reactions at skin incision [21]. One study reported no significant difference in rebound hypertension during the first postoperative week [21].
	Fluid requirement, diuresis	Two studies [7, 23]	One study found an increase in fluid requirements in the clonidine group compared to placebo [5]. Two studies found no significant difference in diuresis between groups [7, 23] and one study found no significant difference in fluid requirements between groups [23].
Respiration	Respiratory rate	Four studies [19, 21, 23, 24]	Three studies reported no significant difference in respiratory rate between groups [19, 21, 23]. One study reported one incidence of respiratory depression in placebo group with no incidence in clonidine group [24].
	Desaturations	Three studies [19, 23, 24]	One study reported 2 incidents of hypoxemia in the clonidine group versus 0 incidents in the control group [24]. One study reported no significant difference in PaO_2_ between the two groups [23]. One study reported no significant difference in SpO_2_ between groups [19]. One study reported no significant difference in number of SpO_2_ < 90% incidents between groups [23]. One study reported 0 arterial desaturations in the intervention group versus 106 arterial desaturations in the control group [23].
	Other respiratory parameters	Two studies [7, 23]	One study found an increased oxygen consumption index in all but one measurement between groups [5]. One study found no significant difference in oxygen requirement between the two groups [23]. Two studies found no significant difference in pH levels or PaCO_2_ [7, 23].
Other	Postoperative nausea and vomiting (PONV)	Five studies [16, 19, 21, 22, 24]	One study found a decrease in incidence of PONV in the clonidine group, but no difference in severity of PONV [22]. One study found a significant decrease in PONV in a group receiving intraoperative and postoperative clonidine compared with a group receiving intraoperative placebo and postoperative morphine [21]. One study reported 4 incidents of PONV in the clonidine group compared to 2 incidents in the control group [24]. Two studies found no significant difference in PONV between groups [16, 19].
	Pharmacokinetics of fentanyl	One study [23]	One study found a decrease in clearance and elimination rate constant of fentanyl [23].
	Body temperature	One study [20]	One study reported no significant difference in the incidence of elevated body temperature in each study group [20].
	Hemoglobin	Two studies [7, 23]	Two studies found no significant difference in blood hemoglobin levels [7, 23].
	Nifedipine administration	One study [5]	One study reported no significant difference in nifedipine administration [5].
	Naloxone requirement	One study [23]	One study found a decrease in naloxone administration [23].
	Hallucinations	Three studies [20, 21, 24]	One study reported 3 incidents of visual symptoms in the clonidine group versus 0 incidents in the control group [24]. Two studies reported no statistical difference in the incidence of hallucinations in any group [20, 21].
	Prolonged ileus and metallic taste	One study [21]	One study reported no incidence of prolonged ileus and metallic taste [21].
	Dreams, pleasant/bad	One study [24]	One study reported two cases of pleasant dreams and 3 cases of bad dreams in the clonidine group versus no pleasant or bad dreams in the control group [24].
	Dizziness, euphoria, and light headed sensation	Two studies [19, 24]	One study found no significant difference in frequency of dizziness and euphoria between the groups [19]. One study reported 9 incidents of light‐headed sensation in the clonidine group versus 1 incidence in the control group [24].
	Cough score	One study [16]	One study found significantly lower median cough scores compared to placebo [16].
	Sleep	One study [17]	One study found a significant increase in sleep duration and quality compared to placebo [17].
	Delirium	One study [17]	One study found no patients having delirium in either group [17].
	Safety outcomes, median HDU‐ and hospital LoS	One study [17]	One study found no significant difference in any safety outcomes, median HDU‐ or hospital LoS [17].
	Plasma clonidine concentration	One study [18]	One study found a dose‐dependent difference in plasma clonidine concentrations [18].

Abbreviations: HDU, High‐Dependency Unit; i.v., intravenous; LoS, Length of Stay; PaCO2, Partial arterial pressure of CO2; PaO2, Partial arterial pressure of O2; pH, power of Hydrogen; PONV, Post‐Operative Nausea and Vomiting; SpO2, oxygen saturation.

Eight trials assessed various hemodynamic parameters [7, 16, 17, 18, 19, 21, 23, 24]. Three out of six trials reporting on heart rate [7, 16, 17, 18, 19, 23] found a significant decrease in the groups receiving clonidine [7, 18, 19]. In all three of these trials, clonidine monotherapy was compared with placebo. Of the other three trials that found no significant difference in heart rate [16, 17, 23], two compared clonidine with placebo [16, 17], while one compared clonidine combined with 25 μg·h^−1^ fentanyl with 75 μg·h^−1^ fentanyl alone [23].

Similarly, four out of seven trials assessing blood pressure reported a statistically significant decrease in the clonidine groups [7, 18, 19, 23]. Of these, three compared clonidine monotherapy with placebo [7, 18, 19], while one compared a fentanyl–clonidine combination with fentanyl alone [23]. The three trials that did not find a significant difference in blood pressure [16, 17, 24] included two trials where clonidine was given as monotherapy compared with placebo [16, 17], and one trial comparing a clonidine–ketamine combination with placebo [24]. Other hemodynamic parameters, including cardiac index and mean pulmonary arterial pressure, generally showed no substantial differences between groups.

Five trials assessed various respiratory parameters including respiratory rate and desaturations [7, 19, 21, 23, 24]. No trials found any statistically significant differences between groups.

Five trials [16, 19, 21, 22, 24] reported on postoperative nausea and vomiting (PONV) and found no substantial differences between groups.

All trials reported on various other adverse effects which are visually depicted in Figure 5.

The certainty of evidence for adverse effects was very low, downgraded for risk of bias, indirectness, and imprecision (Table 2).

## 4 Discussion

In this scoping review, we found that the amount of evidence on intravenous clonidine in postoperative pain management is limited, as the available literature is sparse and the certainty of evidence is very low [13].

Heterogeneity in the timing and dosage of clonidine administration reflects the fact that clonidine is sometimes used as an add‐on in an analgesic regimen and sometimes as a substitute for an opioid. Moreover, the various combinations of other analgesics used across trials further complicate the interpretation of efficacy as well as the safety profile of clonidine. Lastly, variability in the assessment of outcomes such as pain scores, supplemental analgesics, and adverse effects adds to this uncertainty. These differences in interventions and comparators led to serious indirectness for all our outcome measures, which is probably the main concern regarding the quality of the evidence in the included studies.

Our GRADE assessment also revealed serious imprecision for pain outcomes and very serious imprecision for adverse effects. This was mainly caused by low patient numbers, leading to few events, wide confidence intervals, and consequently a considerable risk of Type II errors in the majority of the studies. Lastly, the risk of bias was serious for all outcomes.

This review shows a lack of consistent evidence supporting the use of clonidine in the postoperative setting, as the included trials did not find consistent results supporting favorable effects of clonidine on pain scores and supplemental analgesia. In contrast, a systematic review and meta‐analysis by Blaudszun et al. [25] from 2012 evaluated the effect of perioperative systemic α2‐agonists on postoperative pain and opioid consumption. They found that clonidine significantly reduced cumulative morphine consumption at 24 h by approximately 4 mg and decreased pain scores by 0.7 cm on a 10 cm Visual Analog Scale compared with control groups. Nevertheless, in the study by Blaudszun et al. [25] clonidine was primarily given pre‐ and perioperatively, in contrast to the exclusively postoperative administration studied here.

The optimal dose of intravenous clonidine is not well established. Across the included trials, bolus doses, where used, ranged from 0.5 to 5 μg·kg^−1^ and infusion rates from 0.3 to 2 μg·kg^−1^·h^−1^, and one trial used a fixed dose that was not adjusted for body weight [19]. The trials using the lowest exposures found no effect on pain or supplemental analgesia [17, 24], whereas most of those reporting a benefit used a bolus of at least 3 μg·kg^−1^ [7, 16, 18, 22]. Only one included trial addressed dosing directly: Marinangeli et al. [18] compared boluses of 2, 3 and 5 μg·kg^−1^ followed by an infusion of 0.3 μg·kg^−1^·h^−1^, and found that the reductions in morphine requirement and in systolic blood pressure were both dose‐dependent, proposing 3 μg·kg^−1^ as the best compromise. That analgesia and adverse effects increase in parallel is supported by a trial not eligible for this review, in which patients titrating intravenous clonidine themselves obtained pain relief only at a mean total dose of approximately 800 μg over 10 h, and at the cost of pronounced sedation [26].

What constitutes an optimal dose is therefore likely to depend on the indication, as a degree of sedation that is unacceptable when the aim is opioid‐sparing analgesia may be acceptable, or even desirable, when clonidine is given to attenuate opioid withdrawal or for its anxiolytic and sleep‐promoting properties [17, 27]. A further consideration is that the trials addressing dosing investigated clonidine essentially as monotherapy, with opioids given only as rescue [7, 18]. This design asks whether clonidine can replace an opioid, whereas postoperative pain management is multimodal [2], and the clinically relevant question is whether clonidine adds anything to an established regimen. The dose needed for an additive effect may be lower, as suggested by the trial in which an infusion of 0.3 μg·kg^−1^·h^−1^ combined with one third of the control fentanyl dose gave comparable analgesia with fewer episodes of arterial desaturation [23]. Determining the minimum effective adjunct dose within a standardized multimodal protocol, and for a clearly stated indication, therefore appears more relevant than further study of clonidine as a stand‐alone analgesic.

Nevertheless, it may be premature to dismiss the potential role of postoperative clonidine as part of a multimodal pain strategy, particularly for patients with contraindications to other agents and those with acute‐on‐chronic pain including patients with chronic opioid use. However, for chronic pain patients, most evidence is on non‐systemic routes of administration [28]. In an emergency setting, oral clonidine has been shown to have beneficial effects on pain scores in patients with chronic opioid use suggesting that similar benefits may also occur in the postoperative setting [29]. One smaller study also found a bolus of intravenous clonidine to reduce pain in patients with chronic back pain [30].

Although clonidine is known as an antihypertensive drug [27], the included trials reporting on hemodynamic parameters provided no clear evidence that intravenous clonidine, when given in the doses studied, which ranged from 0.5 to 5 μg·kg^−1^, carries a clinically important risk profile. Three of the four studies reporting decreases in blood pressure [7, 18, 19], compared clonidine with placebo, and two of these three studies also reported reduced pain scores [7, 18]. Therefore, the observed decreases in blood pressure may partly reflect improved pain control rather than representing a clinically relevant adverse hemodynamic effect. Similarly, two studies in this review reported increased sedation in the clonidine groups [16, 18]. Given the analgesic and central sedative properties of clonidine, these findings may also reflect pharmacological effects associated with analgesia rather than clinically concerning adverse effects.

Two systematic reviews evaluated the effect of clonidine on PONV [25, 31] which included five studies that were also assessed in our review [7, 18, 19, 21, 22]. Those reviews included pre‐, peri‐, and postoperative administration of clonidine, and both reviews demonstrated a reduction in early PONV. In contrast, we did not observe a similar effect, which may be due to our narrower focus on postoperative administration, potentially overlooking benefits associated with earlier timing of clonidine administration. Variations in types of surgery and/or patient populations might also contribute to this [32].

For future randomized clinical trials, we recommend focusing on postoperative intravenous clonidine in patients suffering from chronic pain rather than patients with no history of chronic pain, as was the case in most of the studies in this review. In today's clinical practice, this is the patient group in whom other analgesics such as opioids have limited effects [2] and for whom alternative pain management strategies are warranted [33]. In these patients, current strategies are already multimodal [34], and in that setting, clonidine may still have a role to play as an adjunct to standardized pain management strategies.

### 4.1 Limitations

This scoping review has several limitations. Firstly, the heterogeneity of interventions and comparators led to serious indirectness across the included studies, which limits the ability to draw definitive conclusions about the role of clonidine in postoperative pain management. Secondly, we cannot be certain that our search strategy identified all relevant studies. Thirdly, the depth of analysis in a scoping review format limits our ability to evaluate the effects of interventions. Fourthly, data extraction and risk of bias assessment were performed by a single author. Lastly, we included only studies published in English, which may have excluded relevant data, although our search identified only three non‐English studies.

## 5 Conclusion

In this scoping review, we found that studies evaluating the use of intravenous clonidine for postoperative pain management in adult patients were heterogeneous, as reflected by variations in the dosage and timing of clonidine, as well as in the analgesic regimens used as comparators. The reported results suggested little to no effect of clonidine on pain scores or the need for supplemental analgesia, and no clinically important adverse effects were identified; however, the certainty of evidence was very low.

## Author Contributions

All authors meet the ICMJE criteria for authorship. T.M., B.D., and J.H. conceptualized the study. T.M. and E.A.T. conducted the screening of articles. T.M. wrote the original draft. T.M., E.A.T., B.D., M.K., and J.H. reviewed and revised the manuscript. B.D., M.K., and J.H. provided supervision. All authors approved the final manuscript.

## Funding

The authors have nothing to report.

## Conflicts of Interest

The authors declare no conflicts of interest.

## Supporting information


**Table S1:** Preferred reporting items for systematic reviews and meta‐analyses extension for scoping reviews (PRISMA‐ScR) checklist.
**Appendix 1:** Electronic search strategies.
**Table S2:** Data extraction template.
**Table S3:** Details on risk of bias (RoB) assessment using the RoB2 tool.

## Data Availability

Data sharing not applicable to this article as no datasets were generated or analysed during the current study.

## References

[1] D. Wang , Y. Guo , Q. Yin , et al., “Analgesia Quality Index Improves the Quality of Postoperative Pain Management: A Retrospective Observational Study of 14,747 Patients Between 2014 and 2021,” BMC Anesthesiology 23 (2023): 281, 10.1186/s12871-023-02240-8. 37598151 PMC10439647

[2] R. Chou , D. B. Gordon , O. A. de Leon‐Casasola , et al., “Management of Postoperative Pain: A Clinical Practice Guideline From the American Pain Society, the American Society of Regional Anesthesia and Pain Medicine, and the American Society of Anesthesiologists' Committee on Regional Anesthesia, Executive Committee, and Administrative Council,” Journal of Pain 17, no. 2 (2016): 131–157, 10.1016/j.jpain.2015.12.008. 26827847

[3] H. Stähle , “A Historical Perspective: Development of Clonidine,” Best Practice & Research. Clinical Anaesthesiology 14, no. 2 (2000): 237–246, 10.1053/bean.2000.0079.

[4] J. Eisenach , M. De Kock , and W. Klimscha , “α2‐Adrenergic Agonists for Regional Anesthesia: A Clinical Review of Clonidine (1984‐1995),” Anesthesiology 85, no. 3 (1996): 655–674, 10.1097/00000542-199609000-00026. 8853097

[5] A. Pertovaara , “Noradrenergic Pain Modulation,” Progress in Neurobiology 80, no. 2 (2006): 53–83, 10.1016/j.pneurobio.2006.08.001. 17030082

[6] E. Engelman and C. Marsala , “Efficacy of Adding Clonidine to Intrathecal Morphine in Acute Postoperative Pain: Meta‐Analysis,” British Journal of Anaesthesia 110, no. 1 (2013): 21–27, 10.1093/bja/aes344. 23002167

[7] J. M. Bernard , J. L. Hommeril , N. Passuti , and M. Pinaud , “Postoperative Analgesia by Intravenous Clonidine,” Anesthesiology 75, no. 4 (1991): 577–582, 10.1097/00000542-199110000-00006. 1928767

[8] L. Magistris , A. Casati , A. Albertin , G. Aldegheri , and G. Fanelli , “Improving Postoperative Analgesia After Axillary Brachial Plexus Anaesthesia With 0.75% Ropivacaine: A Double‐Blind Evaluation of Adding Clonidine,” European Journal of Anaesthesiology 18, no. Suppl 21 (2001): 75, 10.1097/00003643-200100001-00266. 11382830

[9] W. Erlacher , C. Schuschnig , H. Koinig , et al., “Clonidine as Adjuvant for Mepivacaine, Ropivacaine and Bupivacaine in Axillary, Perivascular Brachial Plexus Block,” Canadian Journal of Anesthesia 48, no. 6 (2001): 522–525, 10.1007/BF03016825. 11444444

[10] S. Ghosh , S. Roy , B. B. Gharami , A. Biswas , D. Bhattacharya , and S. Sen , “A Randomised, Double Blinded Study on Comparison of Intrathecal Morphine and Clonidine as Adjuvant for Post‐Caesarean Analgesia,” Journal of Clinical and Diagnostic Research 14, no. 10 (2020): UC01–UC04, 10.7860/JCDR/2020/45382.14127.

[11] A. C. Tricco , E. Lillie , W. Zarin , et al., “PRISMA Extension for Coping Reviews (PRISMA‐ScR): Checklist and Explanation,” Annals of Internal Medicine 169, no. 7 (2018): 467–473, 10.7326/M18-0850. 30178033

[12] J. A. C. Sterne , J. Savović , M. J. Page , et al., “RoB 2: A Revised Tool for Assessing Risk of Bias in Randomised Trials,” BMJ 366 (2019): l4898, 10.1136/bmj.l4898. 31462531

[13] Z. Al Duhailib , A. Granholm , W. Alhazzani , S. Oczkowski , E. Belley‐Cote , and M. H. Møller , “GRADE Pearls and Pitfalls—Part 1: Systematic Reviews and Meta‐Analyses,” Acta Anaesthesiologica Scandinavica 68, no. 5 (2024): 584–592, 10.1111/aas.14386. 38351600

[14] “GRADE Handbook [Internet],” (2026), https://gdt.gradepro.org/app/handbook/handbook.html.

[15] N. Santesso , C. Glenton , P. Dahm , et al., “GRADE Guidelines 26: Informative Statements to Communicate the Findings of Systematic Reviews of Interventions,” Journal of Clinical Epidemiology 119 (2020): 126–135, 10.1016/j.jclinepi.2019.10.014. 31711912

[16] V. Bedi , S. Goyet , C. M. Thomas , and A. Velloth , “Dexmedetomidine Versus Clonidine for Improved Quality of Emergence From General Anaesthesia: A Randomised Placebo‐Controlled Study,” Journal of Clinical and Diagnostic Research 17, no. 8 (2023): 46–51, 10.7860/JCDR/2023/62586.18353.

[17] D. Liu , E. Hallt , A. Platz , et al., “Low‐Dose Clonidine Infusion to Improve Sleep in Postoperative Patients in the High‐Dependency Unit. A Randomised Placebo‐Controlled Single‐Centre Trial,” Intensive Care Medicine 50, no. 11 (2024): 1873–1883, 10.1007/s00134-024-07619-w. 39311905 PMC11541301

[18] F. Marinangeli , A. Ciccozzi , F. Donatelli , et al., “Clonidine for Treatment of Postoperative Pain: A Dose‐Finding Study,” European Journal of Pain 6, no. 1 (2002): 35–42, 10.1053/eujp.2001.0270. 11888226

[19] W. H. Striebel , D. I. Koenigs , and J. A. Kramer , “Intravenous Clonidine Fails to Reduce Postoperative Meperidine Requirements,” Journal of Clinical Anesthesia 5, no. 3 (1993): 221–225, 10.1016/0952-8180(93)90019-B. 8318241

[20] H. Holthusen , P. Backhaus , F. Boeminghaus , M. Breulmann , and P. Lipfert , “Preemptive Analgesia: No Relevant Advantage of Preoperative Compared With Postoperative Intravenous Administration of Morphine, Ketamine, and Clonidine in Patients Undergoing Transperitoneal Tumor Nephrectomy,” Regional Anesthesia and Pain Medicine 27, no. 3 (2002): 249–253, 10.1053/rapm.2002.30669. 12016597

[21] M. De Kock , P. Lavandhomme , and J. L. Scholtes , “Intraoperative and Postoperative Analgesia Using Intravenous Opioid, Clonidine and Lignocaine,” Anaesthesia and Intensive Care 22, no. 1 (1994): 15–21, 10.1177/0310057X9402200103. 8160943

[22] S. A. Jeffs , J. E. Hall , and S. Morris , “Comparison of Morphine Alone With Morphine Plus Clonidine for Postoperative Patient‐Controlled Analgesia,” British Journal of Anaesthesia 89, no. 3 (2002): 424–427, 10.1093/bja/89.3.424. 12402720

[23] J. M. Bernard , D. Lagarde , and R. Souron , “Balanced Postoperative Analgesia: Effect of Intravenous Clonidine on Blood Gases and Pharmacokinetics of Intravenous Fentanyl,” Anesthesia and Analgesia 79, no. 6 (1994): 1126–1132, 10.1213/00000539-199412000-00018. 7978437

[24] J. C. Salengros , F. Hecquet , K. Touihri , J. Sekkat , L. Barvais , and E. Engelman , “Low‐Dose Intravenous Ketamine and Clonidine for Poor Postoperative Opioid Responsiveness: A Double Blind Randomized Study,” Acta Anaesthesiologica Belgica 62, no. 2 (2011): 65–72. 21919372

[25] G. Blaudszun , C. Lysakowski , N. Elia , and M. R. Tramèr , “Effect of Perioperative Systemic α2 Agonists on Postoperative Morphine Consumption and Pain Intensity: Systematic Review and Meta‐Analysis of Randomized Controlled Trials,” Anesthesiology 116, no. 6 (2012): 1312–1322, 10.1097/ALN.0b013e31825681cb. 22546966

[26] J. M. Bernard , O. Kick , and F. Bonnet , “Comparison of Intravenous and Epidural Clonidine for Postoperative Patient‐Controlled Analgesia,” Anesthesia and Analgesia 81, no. 4 (1995): 706–712, 10.1097/00000539-199510000-00009. 7573998

[27] R. Yasaei and A. Saadabadi , “Clonidine,” in StatPearls [Internet] (StatPearls Publishing, 2025).

[28] A. Kumar , S. Maitra , P. Khanna , and D. K. Baidya , “Clonidine for Management of Chronic Pain: A Brief Review of the Current Evidences,” Saudi Journal of Anaesthesia 8, no. 1 (2014): 92–96, 10.4103/1658-354X.125955. 24665248 PMC3950462

[29] Z. Rostamipoor , M. Nazemi‐Rafi , A. Mirafzal , S. Ilka , and M. Hosseininasab , “Effect of Oral Clonidine on Pain Reduction in Patients With Opioid Use Disorder in the Emergency Department: A Randomized Clinical Trial,” British Journal of Clinical Pharmacology 90, no. 12 (2024): 3003–3009, 10.1111/bcp.15948. 37908055

[30] D. Carroll , A. Jadad , V. King , P. Wiffen , C. Glynn , and H. McQuay , “Single‐Dose, Randomized, Double‐Blind, Double‐Dummy Cross‐Over Comparison of Extradural and i.v. Clonidine in Chronic Pain,” British Journal of Anaesthesia 71, no. 5 (1993): 665–669, 10.1093/bja/71.5.665. 8251276

[31] M. C. Sanchez Munoz , M. De Kock , and P. Forget , “What Is the Place of Clonidine in Anesthesia? Systematic Review and Meta‐Analyses of Randomized Controlled Trials,” Journal of Clinical Anesthesia 38 (2017): 140–153, 10.1016/j.jclinane.2017.02.003. 28372656

[32] M. Komann , Y. Rabe , T. Lehmann , et al., “Operation‐Specific Risk of Postoperative Nausea: A Cross‐Sectional Study Comparing 72 Procedures,” BMJ Open 14, no. 2 (2024): e077508, 10.1136/bmjopen-2023-077508. PMC10882331 38382957

[33] W. Häuser , B. Morlion , K. E. Vowles , et al., “European Clinical Practice Recommendations on Opioids for Chronic Noncancer Pain – Part 1: Role of Opioids in the Management of Chronic Noncancer Pain,” European Journal of Pain 25, no. 5 (2021): 949–968, 10.1002/ejp.1736. 33655607 PMC8248186

[34] R. Dale and B. Stacey , “Multimodal Treatment of Chronic Pain,” Medical Clinics of North America 100, no. 1 (2016): 55–64, 10.1016/j.mcna.2015.08.012. 26614719

