# Supplementary material for: The Use of Intravenous Clonidine in Postoperative Pain Management: A Scoping Review
Source: Acta Anaesthesiol Scand. 2026 Aug 27;70(9):e70329. doi: 10.1111/aas.70329 (PMC13521682; doi:10.1111/aas.70329)
Supplement: Supplementary file 1 [file AAS-70-0-s001.docx]

Supplementary Material

Supplement to: Mohr T, Tsuchiya EA, Krag M, Dybdal B, Heiberg J.

The use of intravenous clonidine in postoperative pain management: A scoping review.

Table of Contents

[Supplementary material 1	3](#_Toc225770318)

[**Table S1 - Preferred Reporting Items for Systematic reviews and Meta-Analyses extension for Scoping Reviews (PRISMA-ScR) Checklist**	3](#_Toc225770319)

[Supplementary material 2	3](#_Toc225770320)

[**Appendix 1**	3](#_Toc225770321)

[Supplementary material 3	4](#_Toc225770322)

[**Table S2 - Data extraction template**	4](#_Toc225770323)

[Supplementary material 4	6](#_Toc225770324)

[**Table S3 -** Details on Risk of Bias (RoB) assessment using the RoB2 tool	6](#_Toc225770325)

# Supplementary material 1

## **Table S1 - Preferred Reporting Items for Systematic reviews and Meta-Analyses extension for Scoping Reviews (PRISMA-ScR) Checklist**

| **SECTION** | **ITEM** | **PRISMA-ScR CHECKLIST ITEM** | **REPORTED ON PAGE #** |
| --- | --- | --- | --- |
| **TITLE** | | | |
| Title | 1 | Identify the report as a scoping review. | Manuscript, p. 1 |
| **ABSTRACT** | | | |
| Structured summary | 2 | Provide a structured summary that includes (as applicable): background, objectives, eligibility criteria, sources of evidence, charting methods, results, and conclusions that relate to the review questions and objectives. | Manuscript, p. 2 |
| **INTRODUCTION** | | | |
| Rationale | 3 | Describe the rationale for the review in the context of what is already known. Explain why the review questions/objectives lend themselves to a scoping review approach. | Manuscript, p. 3 |
| Objectives | 4 | Provide an explicit statement of the questions and objectives being addressed with reference to their key elements (e.g., population or participants, concepts, and context) or other relevant key elements used to conceptualize the review questions and/or objectives. | Manuscript, p. 3 |
| **METHODS** | | | |
| Protocol and registration | 5 | Indicate whether a review protocol exists; state if and where it can be accessed (e.g., a Web address); and if available, provide registration information, including the registration number. | Not relevant. |
| Eligibility criteria | 6 | Specify characteristics of the sources of evidence used as eligibility criteria (e.g., years considered, language, and publication status), and provide a rationale. | Manuscript, p. 4 |
| Information sources* | 7 | Describe all information sources in the search (e.g., databases with dates of coverage and contact with authors to identify additional sources), as well as the date the most recent search was executed. | Manuscript, p. 4 |
| Search | 8 | Present the full electronic search strategy for at least 1 database, including any limits used, such that it could be repeated. | Appendix 1, manuscript, p. 10-11 |
| Selection of sources of evidence† | 9 | State the process for selecting sources of evidence (i.e., screening and eligibility) included in the scoping review. | Manuscript, p. 4 |
| Data charting process‡ | 10 | Describe the methods of charting data from the included sources of evidence (e.g., calibrated forms or forms that have been tested by the team before their use, and whether data charting was done independently or in duplicate) and any processes for obtaining and confirming data from investigators. | Manuscript, p. 4 |
| Data items | 11 | List and define all variables for which data were sought and any assumptions and simplifications made. | Manuscript, p. 5 |
| Critical appraisal of individual sources of evidence§ | 12 | If done, provide a rationale for conducting a critical appraisal of included sources of evidence; describe the methods used and how this information was used in any data synthesis (if appropriate). | Manuscript, p. 5 |
| Synthesis of results | 13 | Describe the methods of handling and summarizing the data that were charted. | Manuscript, p. 4-5 |
| **RESULTS** | | | |
| Selection of sources of evidence | 14 | Give numbers of sources of evidence screened, assessed for eligibility, and included in the review, with reasons for exclusions at each stage, ideally using a flow diagram. | Manuscript, p. 6 and Figure 1 |
| Characteristics of sources of evidence | 15 | For each source of evidence, present characteristics for which data were charted and provide the citations. | Manuscript, p. 6 and Table 1 |
| Critical appraisal within sources of evidence | 16 | If done, present data on critical appraisal of included sources of evidence (see item 12). | Manuscript, p. 7-8, Table 2, and Table S3 |
| Results of individual sources of evidence | 17 | For each included source of evidence, present the relevant data that were charted that relate to the review questions and objectives. | Manuscript, p. 6-8, Table 3, and Table 4. |
| Synthesis of results | 18 | Summarize and/or present the charting results as they relate to the review questions and objectives. | Manuscript p. 6-8 |
| **DISCUSSION** | | | |
| Summary of evidence | 19 | Summarize the main results (including an overview of concepts, themes, and types of evidence available), link to the review questions and objectives, and consider the relevance to key groups. | Manuscript, p. 9-11 |
| Limitations | 20 | Discuss the limitations of the scoping review process. | Manuscript, p. 11 |
| Conclusions | 21 | Provide a general interpretation of the results with respect to the review questions and objectives, as well as potential implications and/or next steps. | Manuscript, p. 11 |
| **FUNDING** | | | |
| Funding | 22 | Describe sources of funding for the included sources of evidence, as well as sources of funding for the scoping review. Describe the role of the funders of the scoping review. | Manuscript, p. 12 |

*From:* Tricco AC, Lillie E, Zarin W, O'Brien KK, Colquhoun H, Levac D, et al. PRISMA Extension for Scoping Reviews (PRISMAScR): Checklist and Explanation. Ann Intern Med. 2018;169:467–473. [doi: 10.7326/M18-0850](http://annals.org/aim/fullarticle/2700389/prisma-extension-scoping-reviews-prisma-scr-checklist-explanation).

# Supplementary material 2

## **Appendix 1**

**Search strategy for CENTRAL (Cochrane Library)**

ID	Search	Hits

#1	((Clonidine or Catapres or Kapvay or Nexiclon or Onyda or Duraclon or Jenloga) NEAR/3 (Intraven*)):ti,ab,kw	330

#2	MeSH descriptor: [Acute Pain] explode all trees	1375

#3	MeSH descriptor: [Pain, Postoperative] explode all trees	22571

#4	MeSH descriptor: [Pain, Procedural] explode all trees	520

#5	((Surgical* or Postopera* or Post-opera* or Acute* or Recovery) NEAR/3 (Pain or Discomfort or Sore or Ache or Hurt)):ti,ab,kw	66217

#6	#2 or #3 or #4 or #5	66797

#7	#1 and #6	83

**Search strategy for MEDLINE**

Ovid MEDLINE(R) ALL <1946 to November 24, 2025>

1	(exp Administration, Intravenous/ or intravenou*.mp. or intra-venous*.mp.) and Clonidine/	1296

2	((Clonidine or Catapres or Kapvay or Nexiclon or Onyda or Duraclon or Jenloga) adj3 (Intravenou* or intra-venous*)).mp.	464

3	1 or 2	1380

4	exp postoperative pain/ or procedural pain/	57580

5	((Surg* or Postopera* or Post-opera* or Acute* or recovery or procedur*) adj3 (Pain or Discomfort or Sore or Ache or Hurt)).mp.	136311

6	4 or 5	138290

7	3 and 6	130

**Search strategy for EMBASE**

Embase <1974 to 2025 November 21>

1	(exp Administration, Intravenous/ or intravenou*.mp. or intra-venous*.mp.) and Clonidine/	9138

2	((Clonidine or Catapres or Kapvay or Nexiclon or Onyda or Duraclon or Jenloga) adj3 (Intravenou* or intra-venous*)).mp.	584

3	1 or 2	9144

4	exp postoperative pain/ or procedural pain/	120016

5	((Surg* or Postopera* or Post-opera* or Acute* or recovery or procedur*) adj3 (Pain or Discomfort or Sore or Ache or Hurt)).mp.	224122

6	4 or 5	224122

7	3 and 6	1432

# Supplementary material 3

## **Table S2 - Data extraction template**

| **Study identification** | | | | | | | | |  |
| --- | --- | --- | --- | --- | --- | --- | --- | --- | --- |
| **Screening ID** | **Author** | | **Title** | **Publication year** |  |  |  |  |  |
|  |  | |  |  |  |  |  |  |  |
| **Study characteristics** | | | | | | | | |  |
| **Study year** | | **Country** | **Design** | **Surgical procedure** | **Setting** | **Intervention** | **Comparator** |  |  |
|  | |  |  |  |  |  |  |  |  |
| **Population characteristics** | | | | | | | | |  |
| **Number of patients** | | **ASA score** | **Age** | **Sex** |  |  |  |  |  |
|  | |  |  |  |  |  |  |  |  |
| **Intervention** | |  |  |  |  |  |  | **Co-Intervention** |  |
| **Number of patients** | | **Medication** | **Dose** | **Timing** | **Administration** |  |  | **Other medication administered** |  |
|  | |  |  |  |  |  |  |  |  |
| **Comparator** | | | | | | | | |  |
| **Number of patients** | **Medication/**  **Placebo** | | **Dose** | **Timing** | **Administration** |  |  |  |  |
|  |  | |  |  |  |  |  |  |  |
| **Outcomes** | | | | **Adverse effects** | | | | |  |
| **Pain assessment strategy** | **Pain scores** | | **Supplemental analgesia strategy** | **Sedation** | **Hemodynamics** | **Respiration** | **Other** | **Any other outcomes** |  |
|  |  | |  |  |  |  |  |  |  |
| **Key findings** | **Other comments** | |  |  |  |  |  |  |  |
|  |  | |  |  |  |  |  |  |  |
|  |  | |  |  |  |  |  |  |  |

# Supplementary material 4

## **Table S3 -** Details on Risk of Bias (RoB) assessment using the RoB2 tool

**Study: Bedi, 2023^16^**

| **Risk of bias arising from the randomization process (domain 1)** | |
| --- | --- |
| **1.1 Was the allocation sequence random?**  General evaluation | **Yes (Y) / Probably Yes (PY) / Probably No (PN) / No (N) / No Information (NI)**  Y |
| **1.2 Was the allocation sequence concealed until participants were enrolled and assigned to interventions?**  General evaluation | **Yes (Y) / Probably Yes (PY) / Probably No (PN) / No (N) / No Information (NI)**    Y |
| **1.3 Did baseline differences between intervention groups suggest a problem with the randomization process?**  General evaluation | **Yes (Y) / Probably Yes (PY) / Probably No (PN) / No (N) / No Information (NI)**   N |
| **Risk-of-bias judgement (domain 1)**  Pain scores  Adverse effects | **Low / High / Some concerns**  Low  Low |
| **Risk of bias due to deviations from the intended interventions of assignment to intervention (domain 2)** | |
| **2.1 Were participants aware of their assigned intervention during the trial?**  General evaluation | **Yes (Y) / Probably Yes (PY) / Probably No (PN) / No (N) / No Information (NI)**  N |
| **2.2 Were carers and people delivering the interventions aware of participants’ assigned intervention during the trial?**  General evaluation | **Yes (Y) / Probably Yes (PY) / Probably No (PN) / No (N) / No Information (NI)**   N |
| **2.3 If Y/PY/NI to 2.1 or 2.2: Were there deviations from the intended intervention that arose because of the trial context?**  Pain scores  Adverse effects | **Not applicable (NA) / Yes (Y) / Probably Yes (PY) / Probably No (PN) / No (N) /No Information (NI)**  NA  NA |
| **2.4 If Y/PY to 2.3: Were these deviations likely to have affected the outcome?**  Pain scores  Adverse effects | **Not applicable (NA) / Yes (Y) / Probably Yes (PY) / Probably No (PN) / No (N) /No Information (NI)**  NA  NA |
| **2.5 If Y/PY/NI to 2.4: Were these deviations from intended intervention balanced between groups?**  Pain scores  Adverse effects | **Not applicable (NA) / Yes (Y) / Probably Yes (PY) / Probably No (PN) / No (N) /No Information (NI)**  NA  NA |
| **2.6 Was an appropriate analysis used to estimate the effect of assignment to intervention?**  Pain scores  Adverse effects | **Yes (Y) / Probably Yes (PY) / Probably No (PN) / No (N) / No Information (NI)**  Y  Y |
| **2.7 If N/PN/NI to 2.6: Was there potential for a substantial impact (on the result) of the failure to analyse participants in the group to which they were randomized?**  Pain scores  Adverse effects | **Not applicable (NA) / Yes (Y) / Probably Yes (PY) / Probably No (PN) / No (N) /No Information (NI)**  NA  NA |
| **Risk-of-bias judgement (domain 2)**  Pain scores  Adverse effects | **Low / High / Some concerns**  Low  Low |
| **Risk of bias due to missing outcome data (domain 3)** |  |
| **3.1 Were data for this outcome available for all, or nearly all, participants randomized?**  Pain scores  Adverse effects | **Yes (Y) / Probably Yes (PY) / Probably No (PN) / No (N) / No Information (NI)**  Y  Y |
| **3.2 If N/PN/NI to 3.1: Is there evidence that the result was not biased by missing outcome data?**  Pain scores  Adverse effects | **Not applicable (NA) / Yes (Y) / Probably Yes (PY) / Probably No (PN) / No (N) /No Information (NI)**  NA  NA |
| **3.3 If N/PN to 3.2: Could missingness in the outcome depend on its true value?**  Pain scores  Adverse effects | **Not applicable (NA) / Yes (Y) / Probably Yes (PY) / Probably No (PN) / No (N) /No Information (NI)**  NA  NA |
| **3.4 If Y/PY/NI to 3.3: Is it likely that missingness in the outcome depended on its true value?**  Pain scores  Adverse effects | **Not applicable (NA) / Yes (Y) / Probably Yes (PY) / Probably No (PN) / No (N) /No Information (NI)**  NA  NA |
| **Risk-of-bias judgement (domain 3)**  Pain scores  Adverse effects | **Low / High / Some concerns**  Low  Low |
| **Risk of bias in measurement of the outcome (domain 4)** | |
| **4.1 Was the method of measuring the outcome inappropriate?**  Pain scores  Adverse effects | **Yes (Y) / Probably Yes (PY) / Probably No (PN) / No (N) / No Information (NI)**  N  N |
| **4.2 Could measurement or ascertainment of the outcome have differed between intervention groups?**  Pain scores  Adverse effects | **Yes (Y) / Probably Yes (PY) / Probably No (PN) / No (N) / No Information (NI)**  N  PN |
| **4.3 If N/PN/NI to 4.1 or 4.2: Were outcome assessors aware of the intervention received by study participants?**  Pain scores  Adverse effects | **Not applicable (NA) / Yes (Y) / Probably Yes (PY) / Probably No (PN) / No (N) /No Information (NI)**  N  N |
| **4.4 If Y/PY/NI to 4.3: Could assessment of the outcome have been influenced by knowledge of intervention received?**  Pain scores  Adverse effects | **Not applicable (NA) / Yes (Y) / Probably Yes (PY) / Probably No (PN) / No (N) /No Information (NI)**  NA  NA |
| **4.5 If Y/PY/NI to 4.4: Is it likely that assessment of the outcome was influenced by knowledge of intervention received?**  Pain scores  Adverse effects | **Not applicable (NA) / Yes (Y) / Probably Yes (PY) / Probably No (PN) / No (N) /No Information (NI)**  NA  NA |
| **Risk-of-bias judgement (domain 4)**  Pain scores  Adverse effects | **Low / High / Some concerns**  Low  Low |
| **Risk of bias in selection of the reported result (domain 5)** |  |
| **5.1 Were the data that produced this result analysed in accordance with a pre-specified analysis plan that was finalized before unblinded outcome data were available for analysis?**  Pain scores  Adverse effects | **Yes (Y) / Probably Yes (PY) / Probably No (PN) / No (N) / No Information (NI)**    Y  PY |
| **5.2 Is the numerical result being assessed likely to have been selected, on the basis of the results, from multiple eligible outcome measurements (e.g. scales, definitions, time points) within the outcome domain?**  Pain scores  Adverse effects | **Yes (Y) / Probably Yes (PY) / Probably No (PN) / No (N) / No Information (NI)**    PN  PN |
| **5.3 Is the numerical result being assessed likely to have been selected, on the basis of the results, from multiple eligible analyses of the data?**  Pain scores  Adverse effects | **Yes (Y) / Probably Yes (PY) / Probably No (PN) / No (N) / No Information (NI)**   PN  PN |
| **Risk-of-bias judgement (domain 5)**  Pain scores  Adverse effects | **Low / High / Some concerns**  Low  Low |
| **Overall risk of bias based on the previous domains (domain 6)** |  |
| **Overall risk of bias**  Pain scores  Adverse effects | **Low / High / Some concerns**  Low  Low |

**Study: Bernard, 1991^7^**

| **Risk of bias arising from the randomization process (domain 1)** | |
| --- | --- |
| **1.1 Was the allocation sequence random?**  General evaluation | **Yes (Y) / Probably Yes (PY) / Probably No (PN) / No (N) / No Information (NI)**  NI |
| **1.2 Was the allocation sequence concealed until participants were enrolled and assigned to interventions?**  General evaluation | **Yes (Y) / Probably Yes (PY) / Probably No (PN) / No (N) / No Information (NI)**   NI |
| **1.3 Did baseline differences between intervention groups suggest a problem with the randomization process?**  General evaluation | **Yes (Y) / Probably Yes (PY) / Probably No (PN) / No (N) / No Information (NI)**  PN |
| **Risk-of-bias judgement (domain 1)**  Pain scores  Supplemental analgesia  Adverse effects | **Low / High / Some concerns**  Some concerns  Some concerns  Some concerns |
| **Risk of bias due to deviations from the intended interventions of assignment to intervention (domain 2)** | |
| **2.1 Were participants aware of their assigned intervention during the trial?**  General evaluation | **Yes (Y) / Probably Yes (PY) / Probably No (PN) / No (N) / No Information (NI)**  PN |
| **2.2 Were carers and people delivering the interventions aware of participants’ assigned intervention during the trial?**  General evaluation | **Yes (Y) / Probably Yes (PY) / Probably No (PN) / No (N) / No Information (NI)**   PN |
| **2.3 If Y/PY/NI to 2.1 or 2.2: Were there deviations from the intended intervention that arose because of the trial context?**  Pain scores  Supplemental analgesia  Adverse effects | **Not applicable (NA) / Yes (Y) / Probably Yes (PY) / Probably No (PN) / No (N) /No Information (NI)**  NA  NA  NA |
| **2.4 If Y/PY to 2.3: Were these deviations likely to have affected the outcome?**  Pain scores  Supplemental analgesia  Adverse effects | **Not applicable (NA) / Yes (Y) / Probably Yes (PY) / Probably No (PN) / No (N) /No Information (NI)**  NA  NA  NA |
| **2.5 If Y/PY/NI to 2.4: Were these deviations from intended intervention balanced between groups?**  Pain scores  Supplemental analgesia  Adverse effects | **Not applicable (NA) / Yes (Y) / Probably Yes (PY) / Probably No (PN) / No (N) /No Information (NI)**  NA  NA  NA |
| **2.6 Was an appropriate analysis used to estimate the effect of assignment to intervention?**  Pain scores  Supplemental analgesia  Adverse effects | **Yes (Y) / Probably Yes (PY) / Probably No (PN) / No (N) / No Information (NI)**  PY  PY  PY |
| **2.7 If N/PN/NI to 2.6: Was there potential for a substantial impact (on the result) of the failure to analyse participants in the group to which they were randomized?**  Pain scores  Supplemental analgesia  Adverse effects | **Not applicable (NA) / Yes (Y) / Probably Yes (PY) / Probably No (PN) / No (N) /No Information (NI)**  NA  NA  NA |
| **Risk-of-bias judgement (domain 2)**  Pain scores  Supplemental analgesia  Adverse effects | **Low / High / Some concerns**  Low  Low  Low |
| **Risk of bias due to missing outcome data (domain 3)** |  |
| **3.1 Were data for this outcome available for all, or nearly all, participants randomized?**  Pain scores  Supplemental analgesia  Adverse effects | **Yes (Y) / Probably Yes (PY) / Probably No (PN) / No (N) / No Information (NI)**  PY  Y  PY |
| **3.2 If N/PN/NI to 3.1: Is there evidence that the result was not biased by missing outcome data?**  Pain scores  Supplemental analgesia  Adverse effects | **Not applicable (NA) / Yes (Y) / Probably Yes (PY) / Probably No (PN) / No (N) /No Information (NI)**  NA  NA  NA |
| **3.3 If N/PN to 3.2: Could missingness in the outcome depend on its true value?**  Pain scores  Supplemental analgesia  Adverse effects | **Not applicable (NA) / Yes (Y) / Probably Yes (PY) / Probably No (PN) / No (N) /No Information (NI)**   NA  NA  NA |
| **3.4 If Y/PY/NI to 3.3: Is it likely that missingness in the outcome depended on its true value?**  Pain scores  Supplemental analgesia  Adverse effects | **Not applicable (NA) / Yes (Y) / Probably Yes (PY) / Probably No (PN) / No (N) /No Information (NI)**  NA  NA  NA |
| **Risk-of-bias judgement (domain 3)**  Pain scores  Supplemental analgesia  Adverse effects | **Low / High / Some concerns**  Low  Low  Low |
| **Risk of bias in measurement of the outcome (domain 4)** | |
| **4.1 Was the method of measuring the outcome inappropriate?**  Pain scores  Supplemental analgesia  Adverse effects | **Yes (Y) / Probably Yes (PY) / Probably No (PN) / No (N) / No Information (NI)**  N  PN  PN |
| **4.2 Could measurement or ascertainment of the outcome have differed between intervention groups?**  Pain scores  Supplemental analgesia  Adverse effects | **Yes (Y) / Probably Yes (PY) / Probably No (PN) / No (N) / No Information (NI)**  N  N  PN |
| **4.3 If N/PN/NI to 4.1 or 4.2: Were outcome assessors aware of the intervention received by study participants?**  Pain scores  Supplemental analgesia  Adverse effects | **Not applicable (NA) / Yes (Y) / Probably Yes (PY) / Probably No (PN) / No (N) /No Information (NI)**  PN  PN  PN |
| **4.4 If Y/PY/NI to 4.3: Could assessment of the outcome have been influenced by knowledge of intervention received?**  Pain scores  Supplemental analgesia  Adverse effects | **Not applicable (NA) / Yes (Y) / Probably Yes (PY) / Probably No (PN) / No (N) /No Information (NI)**  NA  NA  NA |
| **4.5 If Y/PY/NI to 4.4: Is it likely that assessment of the outcome was influenced by knowledge of intervention received?**  Pain scores  Supplemental analgesia  Adverse effects | **Not applicable (NA) / Yes (Y) / Probably Yes (PY) / Probably No (PN) / No (N) /No Information (NI)**  NA  NA  NA |
| **Risk-of-bias judgement (domain 4)**  Pain scores  Supplemental analgesia  Adverse effects | **Low / High / Some concerns**  Low  Low  Low |
| **Risk of bias in selection of the reported result (domain 5)** |  |
| **5.1 Were the data that produced this result analysed in accordance with a pre-specified analysis plan that was finalized before unblinded outcome data were available for analysis?**  Pain scores  Supplemental analgesia  Adverse effects | **Yes (Y) / Probably Yes (PY) / Probably No (PN) / No (N) / No Information (NI)**    NI  NI  NI |
| **5.2 Is the numerical result being assessed likely to have been selected, on the basis of the results, from multiple eligible outcome measurements (e.g. scales, definitions, time points) within the outcome domain?**  Pain scores  Supplemental analgesia  Adverse effects | **Yes (Y) / Probably Yes (PY) / Probably No (PN) / No (N) / No Information (NI)**    PN  PN  PN |
| **5.3 Is the numerical result being assessed likely to have been selected, on the basis of the results, from multiple eligible analyses of the data?**  Pain scores  Supplemental analgesia  Adverse effects | **Yes (Y) / Probably Yes (PY) / Probably No (PN) / No (N) / No Information (NI)**  PN  PN  PN |
| **Risk-of-bias judgement (domain 5)**  Pain scores  Supplemental analgesia  Adverse effects | **Low / High / Some concerns**  Some concerns  Some concerns  Some concerns |
| **Overall risk of bias based on the previous domains (domain 6)** |  |
| **Overall risk of bias**  Pain scores  Supplemental analgesia  Adverse effects | **Low / High / Some concerns**  Some concerns  Some concerns  Some concerns |

**Study: Bernard, 1994^23^**

| **Risk of bias arising from the randomization process (domain 1)** | |
| --- | --- |
| **1.1 Was the allocation sequence random?**  General evaluation | **Yes (Y) / Probably Yes (PY) / Probably No (PN) / No (N) / No Information (NI)**  NI |
| **1.2 Was the allocation sequence concealed until participants were enrolled and assigned to interventions?**  General evaluation | **Yes (Y) / Probably Yes (PY) / Probably No (PN) / No (N) / No Information (NI)**   NI |
| **1.3 Did baseline differences between intervention groups suggest a problem with the randomization process?**  General evaluation | **Yes (Y) / Probably Yes (PY) / Probably No (PN) / No (N) / No Information (NI)**  N |
| **Risk-of-bias judgement (domain 1)**  Pain scores  Supplemental analgesia  Adverse effects | **Low / High / Some concerns**  Some concerns  Some concerns  Some concerns |
| **Risk of bias due to deviations from the intended interventions of assignment to intervention (domain 2)** | |
| **2.1 Were participants aware of their assigned intervention during the trial?**  General evaluation | **Yes (Y) / Probably Yes (PY) / Probably No (PN) / No (N) / No Information (NI)**  No |
| **2.2 Were carers and people delivering the interventions aware of participants’ assigned intervention during the trial?**  General evaluation | **Yes (Y) / Probably Yes (PY) / Probably No (PN) / No (N) / No Information (NI)**   No |
| **2.3 If Y/PY/NI to 2.1 or 2.2: Were there deviations from the intended intervention that arose because of the trial context?**  Pain scores  Supplemental analgesia  Adverse effects | **Not applicable (NA) / Yes (Y) / Probably Yes (PY) / Probably No (PN) / No (N) /No Information (NI)**  NA  NA  NA |
| **2.4 If Y/PY to 2.3: Were these deviations likely to have affected the outcome?**  Pain scores  Supplemental analgesia  Adverse effects | **Not applicable (NA) / Yes (Y) / Probably Yes (PY) / Probably No (PN) / No (N) /No Information (NI)**   NA  NA  NA |
| **2.5 If Y/PY/NI to 2.4: Were these deviations from intended intervention balanced between groups?**  Pain scores  Supplemental analgesia  Adverse effects | **Not applicable (NA) / Yes (Y) / Probably Yes (PY) / Probably No (PN) / No (N) /No Information (NI)**  NA  NA  NA |
| **2.6 Was an appropriate analysis used to estimate the effect of assignment to intervention?**  Pain scores  Supplemental analgesia  Adverse effects | **Yes (Y) / Probably Yes (PY) / Probably No (PN) / No (N) / No Information (NI)**  N  N  N |
| **2.7 If N/PN/NI to 2.6: Was there potential for a substantial impact (on the result) of the failure to analyse participants in the group to which they were randomized?**  Pain scores  Supplemental analgesia  Adverse effects | **Not applicable (NA) / Yes (Y) / Probably Yes (PY) / Probably No (PN) / No (N) /No Information (NI)**  Y  Y  PN |
| **Risk-of-bias judgement (domain 2)**  Pain scores  Supplemental analgesia  Adverse effects | **Low / High / Some concerns**  High  High  Some concerns |
| **Risk of bias due to missing outcome data (domain 3)** |  |
| **3.1 Were data for this outcome available for all, or nearly all, participants randomized?**  Pain scores  Supplemental analgesia  Adverse effects | **Yes (Y) / Probably Yes (PY) / Probably No (PN) / No (N) / No Information (NI)**  N  N  N |
| **3.2 If N/PN/NI to 3.1: Is there evidence that the result was not biased by missing outcome data?**  Pain scores  Supplemental analgesia  Adverse effects | **Not applicable (NA) / Yes (Y) / Probably Yes (PY) / Probably No (PN) / No (N) /No Information (NI)**  N  PN  N |
| **3.3 If N/PN to 3.2: Could missingness in the outcome depend on its true value?**  Pain scores  Supplemental analgesia  Adverse effects | **Not applicable (NA) / Yes (Y) / Probably Yes (PY) / Probably No (PN) / No (N) /No Information (NI)**   Y  Y  Y |
| **3.4 If Y/PY/NI to 3.3: Is it likely that missingness in the outcome depended on its true value?**  Pain scores  Supplemental analgesia  Adverse effects | **Not applicable (NA) / Yes (Y) / Probably Yes (PY) / Probably No (PN) / No (N) /No Information (NI)**  Y  Y  PN |
| **Risk-of-bias judgement (domain 3)**  Pain scores  Supplemental analgesia  Adverse effects | **Low / High / Some concerns**  High  High  Low |
| **Risk of bias in measurement of the outcome (domain 4)** | |
| **4.1 Was the method of measuring the outcome inappropriate?**  Pain scores  Supplemental analgesia  Adverse effects | **Yes (Y) / Probably Yes (PY) / Probably No (PN) / No (N) / No Information (NI)**  N  N  PN |
| **4.2 Could measurement or ascertainment of the outcome have differed between intervention groups?**  Pain scores  Supplemental analgesia  Adverse effects | **Yes (Y) / Probably Yes (PY) / Probably No (PN) / No (N) / No Information (NI)**  N  N  N |
| **4.3 If N/PN/NI to 4.1 or 4.2: Were outcome assessors aware of the intervention received by study participants?**  Pain scores  Supplemental analgesia  Adverse effects | **Not applicable (NA) / Yes (Y) / Probably Yes (PY) / Probably No (PN) / No (N) /No Information (NI)**  N  PN  PN |
| **4.4 If Y/PY/NI to 4.3: Could assessment of the outcome have been influenced by knowledge of intervention received?**  Pain scores  Supplemental analgesia  Adverse effects | **Not applicable (NA) / Yes (Y) / Probably Yes (PY) / Probably No (PN) / No (N) /No Information (NI)**  NA  NA  NA |
| **4.5 If Y/PY/NI to 4.4: Is it likely that assessment of the outcome was influenced by knowledge of intervention received?**  Pain scores  Supplemental analgesia  Adverse effects | **Not applicable (NA) / Yes (Y) / Probably Yes (PY) / Probably No (PN) / No (N) /No Information (NI)**  NA  NA  NA |
| **Risk-of-bias judgement (domain 4)**  Pain scores  Supplemental analgesia  Adverse effects | **Low / High / Some concerns**  Low  Low  Low |
| **Risk of bias in selection of the reported result (domain 5)** |  |
| **5.1 Were the data that produced this result analysed in accordance with a pre-specified analysis plan that was finalized before unblinded outcome data were available for analysis?**  Pain scores  Supplemental analgesia  Adverse effects | **Yes (Y) / Probably Yes (PY) / Probably No (PN) / No (N) / No Information (NI)**    NI  NI  NI |
| **5.2 Is the numerical result being assessed likely to have been selected, on the basis of the results, from multiple eligible outcome measurements (e.g. scales, definitions, time points) within the outcome domain?**  Pain scores  Supplemental analgesia  Adverse effects | **Yes (Y) / Probably Yes (PY) / Probably No (PN) / No (N) / No Information (NI)**    PN  PN  PN |
| **5.3 Is the numerical result being assessed likely to have been selected, on the basis of the results, from multiple eligible analyses of the data?**  Pain scores  Supplemental analgesia  Adverse effects | **Yes (Y) / Probably Yes (PY) / Probably No (PN) / No (N) / No Information (NI)**  PN  PN  PN |
| **Risk-of-bias judgement (domain 5)**  Pain scores  Supplemental analgesia  Adverse effects | **Low / High / Some concerns**  Some concerns  Some concerns  Some concerns |
| **Overall risk of bias based on the previous domains (domain 6)** |  |
| **Overall risk of bias**  Pain scores  Supplemental analgesia  Adverse effects | **Low / High / Some concerns**  High  High  Some concerns |

**Study: DeKock, 1994^21^**

| **Risk of bias arising from the randomization process (domain 1)** | |
| --- | --- |
| **1.1 Was the allocation sequence random?**  General evaluation | **Yes (Y) / Probably Yes (PY) / Probably No (PN) / No (N) / No Information (NI)**  NI |
| **1.2 Was the allocation sequence concealed until participants were enrolled and assigned to interventions?**  General evaluation | **Yes (Y) / Probably Yes (PY) / Probably No (PN) / No (N) / No Information (NI)**   NI |
| **1.3 Did baseline differences between intervention groups suggest a problem with the randomization process?**  General evaluation | **Yes (Y) / Probably Yes (PY) / Probably No (PN) / No (N) / No Information (NI)**   N |
| **Risk-of-bias judgement (domain 1)**  Pain scores  Supplemental analgesia  Adverse effects | **Low / High / Some concerns**  Some concerns  Some concerns  Some concerns |
| **Risk of bias due to deviations from the intended interventions of assignment to intervention (domain 2)** | |
| **2.1 Were participants aware of their assigned intervention during the trial?**  General evaluation | **Yes (Y) / Probably Yes (PY) / Probably No (PN) / No (N) / No Information (NI)**  N |
| **2.2 Were carers and people delivering the interventions aware of participants’ assigned intervention during the trial?**  General evaluation | **Yes (Y) / Probably Yes (PY) / Probably No (PN) / No (N) / No Information (NI)**   Y |
| **2.3 If Y/PY/NI to 2.1 or 2.2: Were there deviations from the intended intervention that arose because of the trial context?**  Pain scores  Supplemental analgesia  Adverse effects | **Not applicable (NA) / Yes (Y) / Probably Yes (PY) / Probably No (PN) / No (N) /No Information (NI)**  N  N  N |
| **2.4 If Y/PY to 2.3: Were these deviations likely to have affected the outcome?**  Pain scores  Supplemental analgesia  Adverse effects | **Not applicable (NA) / Yes (Y) / Probably Yes (PY) / Probably No (PN) / No (N) /No Information (NI)**   NA  NA  NA |
| **2.5 If Y/PY/NI to 2.4: Were these deviations from intended intervention balanced between groups?**  Pain scores  Supplemental analgesia  Adverse effects | **Not applicable (NA) / Yes (Y) / Probably Yes (PY) / Probably No (PN) / No (N) /No Information (NI)**  NA  NA  NA |
| **2.6 Was an appropriate analysis used to estimate the effect of assignment to intervention?**  Pain scores  Supplemental analgesia  Adverse effects | **Yes (Y) / Probably Yes (PY) / Probably No (PN) / No (N) / No Information (NI)**  PY  PY  PY |
| **2.7 If N/PN/NI to 2.6: Was there potential for a substantial impact (on the result) of the failure to analyse participants in the group to which they were randomized?**  Pain scores  Supplemental analgesia  Adverse effects | **Not applicable (NA) / Yes (Y) / Probably Yes (PY) / Probably No (PN) / No (N) /No Information (NI)**  NA  NA  NA |
| **Risk-of-bias judgement (domain 2)**  Pain scores  Supplemental analgesia  Adverse effects | **Low / High / Some concerns**  Low  Low  Low |
| **Risk of bias due to missing outcome data (domain 3)** |  |
| **3.1 Were data for this outcome available for all, or nearly all, participants randomized?**  Pain scores  Supplemental analgesia  Adverse effects | **Yes (Y) / Probably Yes (PY) / Probably No (PN) / No (N) / No Information (NI)**  Y  Y  Y |
| **3.2 If N/PN/NI to 3.1: Is there evidence that the result was not biased by missing outcome data?**  Pain scores  Supplemental analgesia  Adverse effects | **Not applicable (NA) / Yes (Y) / Probably Yes (PY) / Probably No (PN) / No (N) /No Information (NI)**  NA  NA  NA |
| **3.3 If N/PN to 3.2: Could missingness in the outcome depend on its true value?**  Pain scores  Supplemental analgesia  Adverse effects | **Not applicable (NA) / Yes (Y) / Probably Yes (PY) / Probably No (PN) / No (N) /No Information (NI)**   NA  NA  NA |
| **3.4 If Y/PY/NI to 3.3: Is it likely that missingness in the outcome depended on its true value?**  Pain scores  Supplemental analgesia  Adverse effects | **Not applicable (NA) / Yes (Y) / Probably Yes (PY) / Probably No (PN) / No (N) /No Information (NI)**  NA  NA  NA |
| **Risk-of-bias judgement (domain 3)**  Pain scores  Supplemental analgesia  Adverse effects | **Low / High / Some concerns**  Low  Low  Low |
| **Risk of bias in measurement of the outcome (domain 4)** | |
| **4.1 Was the method of measuring the outcome inappropriate?**  Pain scores  Supplemental analgesia  Adverse effects | **Yes (Y) / Probably Yes (PY) / Probably No (PN) / No (N) / No Information (NI)**  PN  N  N |
| **4.2 Could measurement or ascertainment of the outcome have differed between intervention groups?**  Pain scores  Supplemental analgesia  Adverse effects | **Yes (Y) / Probably Yes (PY) / Probably No (PN) / No (N) / No Information (NI)**  N  N  N |
| **4.3 If N/PN/NI to 4.1 or 4.2: Were outcome assessors aware of the intervention received by study participants?**  Pain scores  Supplemental analgesia  Adverse effects | **Not applicable (NA) / Yes (Y) / Probably Yes (PY) / Probably No (PN) / No (N) /No Information (NI)**  N  N  N |
| **4.4 If Y/PY/NI to 4.3: Could assessment of the outcome have been influenced by knowledge of intervention received?**  Pain scores  Supplemental analgesia  Adverse effects | **Not applicable (NA) / Yes (Y) / Probably Yes (PY) / Probably No (PN) / No (N) /No Information (NI)**  NA  NA  NA |
| **4.5 If Y/PY/NI to 4.4: Is it likely that assessment of the outcome was influenced by knowledge of intervention received?**  Pain scores  Supplemental analgesia  Adverse effects | **Not applicable (NA) / Yes (Y) / Probably Yes (PY) / Probably No (PN) / No (N) /No Information (NI)**  NA  NA  NA |
| **Risk-of-bias judgement (domain 4)**  Pain scores  Supplemental analgesia  Adverse effects | **Low / High / Some concerns**  Low  Low  Low |
| **Risk of bias in selection of the reported result (domain 5)** |  |
| **5.1 Were the data that produced this result analysed in accordance with a pre-specified analysis plan that was finalized before unblinded outcome data were available for analysis?**  Pain scores  Supplemental analgesia  Adverse effects | **Yes (Y) / Probably Yes (PY) / Probably No (PN) / No (N) / No Information (NI)**    NA  NA  NA |
| **5.2 Is the numerical result being assessed likely to have been selected, on the basis of the results, from multiple eligible outcome measurements (e.g. scales, definitions, time points) within the outcome domain?**  Pain scores  Supplemental analgesia  Adverse effects | **Yes (Y) / Probably Yes (PY) / Probably No (PN) / No (N) / No Information (NI)**    N  N  N |
| **5.3 Is the numerical result being assessed likely to have been selected, on the basis of the results, from multiple eligible analyses of the data?**  Pain scores  Supplemental analgesia  Adverse effects | **Yes (Y) / Probably Yes (PY) / Probably No (PN) / No (N) / No Information (NI)**  PN  PN  PN |
| **Risk-of-bias judgement (domain 5)**  Pain scores  Supplemental analgesia  Adverse effects | **Low / High / Some concerns**  Some concerns  Some concerns  Some concerns |
| **Overall risk of bias based on the previous domains (domain 6)** |  |
| **Overall risk of bias**  Pain scores  Supplemental analgesia  Adverse effects | **Low / High / Some concerns**  Some concerns  Some concerns  Some concerns |

**Study: Holthusen, 2002^20^**

| **Risk of bias arising from the randomization process (domain 1)** | |
| --- | --- |
| **1.1 Was the allocation sequence random?**  General evaluation | **Yes (Y) / Probably Yes (PY) / Probably No (PN) / No (N) / No Information (NI)**  NI |
| **1.2 Was the allocation sequence concealed until participants were enrolled and assigned to interventions?**  General evaluation | **Yes (Y) / Probably Yes (PY) / Probably No (PN) / No (N) / No Information (NI)**   NI |
| **1.3 Did baseline differences between intervention groups suggest a problem with the randomization process?**  General evaluation | **Yes (Y) / Probably Yes (PY) / Probably No (PN) / No (N) / No Information (NI)**   PN |
| **Risk-of-bias judgement (domain 1)**  Pain scores  Supplemental analgesia  Adverse effects | **Low / High / Some concerns**  Some concerns  Some concerns  Some concerns |
| **Risk of bias due to deviations from the intended interventions of assignment to intervention (domain 2)** | |
| **2.1 Were participants aware of their assigned intervention during the trial?**  General evaluation | **Yes (Y) / Probably Yes (PY) / Probably No (PN) / No (N) / No Information (NI)**  N |
| **2.2 Were carers and people delivering the interventions aware of participants’ assigned intervention during the trial?**  General evaluation | **Yes (Y) / Probably Yes (PY) / Probably No (PN) / No (N) / No Information (NI)**   N |
| **2.3 If Y/PY/NI to 2.1 or 2.2: Were there deviations from the intended intervention that arose because of the trial context?**  Pain scores  Supplemental analgesia  Adverse effects | **Not applicable (NA) / Yes (Y) / Probably Yes (PY) / Probably No (PN) / No (N) /No Information (NI)**  NA  NA  NA |
| **2.4 If Y/PY to 2.3: Were these deviations likely to have affected the outcome?**  Pain scores  Supplemental analgesia  Adverse effects | **Not applicable (NA) / Yes (Y) / Probably Yes (PY) / Probably No (PN) / No (N) /No Information (NI)**   NA  NA  NA |
| **2.5 If Y/PY/NI to 2.4: Were these deviations from intended intervention balanced between groups?**  Pain scores  Supplemental analgesia  Adverse effects | **Not applicable (NA) / Yes (Y) / Probably Yes (PY) / Probably No (PN) / No (N) /No Information (NI)**  NA  NA  NA |
| **2.6 Was an appropriate analysis used to estimate the effect of assignment to intervention?**  Pain scores  Supplemental analgesia  Adverse effects | **Yes (Y) / Probably Yes (PY) / Probably No (PN) / No (N) / No Information (NI)**  PY  PY  PY |
| **2.7 If N/PN/NI to 2.6: Was there potential for a substantial impact (on the result) of the failure to analyse participants in the group to which they were randomized?**  Pain scores  Supplemental analgesia  Adverse effects | **Not applicable (NA) / Yes (Y) / Probably Yes (PY) / Probably No (PN) / No (N) /No Information (NI)**  NA  NA  NA |
| **Risk-of-bias judgement (domain 2)**  Pain scores  Supplemental analgesia  Adverse effects | **Low / High / Some concerns**  Low  Low  Low |
| **Risk of bias due to missing outcome data (domain 3)** |  |
| **3.1 Were data for this outcome available for all, or nearly all, participants randomized?**  Pain scores  Supplemental analgesia  Adverse effects | **Yes (Y) / Probably Yes (PY) / Probably No (PN) / No (N) / No Information (NI)**  Y  PY  NI |
| **3.2 If N/PN/NI to 3.1: Is there evidence that the result was not biased by missing outcome data?**  Pain scores  Supplemental analgesia  Adverse effects | **Not applicable (NA) / Yes (Y) / Probably Yes (PY) / Probably No (PN) / No (N) /No Information (NI)**  NA  NA  NI |
| **3.3 If N/PN to 3.2: Could missingness in the outcome depend on its true value?**  Pain scores  Supplemental analgesia  Adverse effects | **Not applicable (NA) / Yes (Y) / Probably Yes (PY) / Probably No (PN) / No (N) /No Information (NI)**   NA  NA  PN |
| **3.4 If Y/PY/NI to 3.3: Is it likely that missingness in the outcome depended on its true value?**  Pain scores  Supplemental analgesia  Adverse effects | **Not applicable (NA) / Yes (Y) / Probably Yes (PY) / Probably No (PN) / No (N) /No Information (NI)**  NA  NA  NA |
| **Risk-of-bias judgement (domain 3)**  Pain scores  Supplemental analgesia  Adverse effects | **Low / High / Some concerns**  Low  Low  Low |
| **Risk of bias in measurement of the outcome (domain 4)** | |
| **4.1 Was the method of measuring the outcome inappropriate?**  Pain scores  Supplemental analgesia  Adverse effects | **Yes (Y) / Probably Yes (PY) / Probably No (PN) / No (N) / No Information (NI)**  N  N  PN |
| **4.2 Could measurement or ascertainment of the outcome have differed between intervention groups?**  Pain scores  Supplemental analgesia  Adverse effects | **Yes (Y) / Probably Yes (PY) / Probably No (PN) / No (N) / No Information (NI)**  N  PN  PN |
| **4.3 If N/PN/NI to 4.1 or 4.2: Were outcome assessors aware of the intervention received by study participants?**  Pain scores  Supplemental analgesia  Adverse effects | **Not applicable (NA) / Yes (Y) / Probably Yes (PY) / Probably No (PN) / No (N) /No Information (NI)**  N  N  PN |
| **4.4 If Y/PY/NI to 4.3: Could assessment of the outcome have been influenced by knowledge of intervention received?**  Pain scores  Supplemental analgesia  Adverse effects | **Not applicable (NA) / Yes (Y) / Probably Yes (PY) / Probably No (PN) / No (N) /No Information (NI)**  NA  NA  NA |
| **4.5 If Y/PY/NI to 4.4: Is it likely that assessment of the outcome was influenced by knowledge of intervention received?**  Pain scores  Supplemental analgesia  Adverse effects | **Not applicable (NA) / Yes (Y) / Probably Yes (PY) / Probably No (PN) / No (N) /No Information (NI)**  NA  NA  NA |
| **Risk-of-bias judgement (domain 4)**  Pain scores  Supplemental analgesia  Adverse effects | **Low / High / Some concerns**  Low  Low  Low |
| **Risk of bias in selection of the reported result (domain 5)** |  |
| **5.1 Were the data that produced this result analysed in accordance with a pre-specified analysis plan that was finalized before unblinded outcome data were available for analysis?**  Pain scores  Supplemental analgesia  Adverse effects | **Yes (Y) / Probably Yes (PY) / Probably No (PN) / No (N) / No Information (NI)**    NI  NI  PN |
| **5.2 Is the numerical result being assessed likely to have been selected, on the basis of the results, from multiple eligible outcome measurements (e.g. scales, definitions, time points) within the outcome domain?**  Pain scores  Supplemental analgesia  Adverse effects | **Yes (Y) / Probably Yes (PY) / Probably No (PN) / No (N) / No Information (NI)**    PN  PN  NI |
| **5.3 Is the numerical result being assessed likely to have been selected, on the basis of the results, from multiple eligible analyses of the data?**  Pain scores  Supplemental analgesia  Adverse effects | **Yes (Y) / Probably Yes (PY) / Probably No (PN) / No (N) / No Information (NI)**  PN  PN  NI |
| **Risk-of-bias judgement (domain 5)**  Pain scores  Supplemental analgesia  Adverse effects | **Low / High / Some concerns**  Some concerns  Some concerns  High |
| **Overall risk of bias based on the previous domains (domain 6)** |  |
| **Overall risk of bias**  Pain scores  Supplemental analgesia  Adverse effects | **Low / High / Some concerns**  Some concerns  Some concerns  High |

**Study: Jeffs, 2002^22^**

| **Risk of bias arising from the randomization process (domain 1)** | |
| --- | --- |
| **1.1 Was the allocation sequence random?**  General evaluation | **Yes (Y) / Probably Yes (PY) / Probably No (PN) / No (N) / No Information (NI)**  Y |
| **1.2 Was the allocation sequence concealed until participants were enrolled and assigned to interventions?**  General evaluation | **Yes (Y) / Probably Yes (PY) / Probably No (PN) / No (N) / No Information (NI)**   Y |
| **1.3 Did baseline differences between intervention groups suggest a problem with the randomization process?**  General evaluation | **Yes (Y) / Probably Yes (PY) / Probably No (PN) / No (N) / No Information (NI)**   N |
| **Risk-of-bias judgement (domain 1)**  Pain scores  Supplemental analgesia  Adverse effects | **Low / High / Some concerns**  Low  Low  Low |
| **Risk of bias due to deviations from the intended interventions of assignment to intervention (domain 2)** | |
| **2.1 Were participants aware of their assigned intervention during the trial?**  General evaluation | **Yes (Y) / Probably Yes (PY) / Probably No (PN) / No (N) / No Information (NI)**  N |
| **2.2 Were carers and people delivering the interventions aware of participants’ assigned intervention during the trial?**  General evaluation | **Yes (Y) / Probably Yes (PY) / Probably No (PN) / No (N) / No Information (NI)**   NI |
| **2.3 If Y/PY/NI to 2.1 or 2.2: Were there deviations from the intended intervention that arose because of the trial context?**  Pain scores  Supplemental analgesia  Adverse effects | **Not applicable (NA) / Yes (Y) / Probably Yes (PY) / Probably No (PN) / No (N) /No Information (NI)**  PN  PN  PN |
| **2.4 If Y/PY to 2.3: Were these deviations likely to have affected the outcome?**  Pain scores  Supplemental analgesia  Adverse effects | **Not applicable (NA) / Yes (Y) / Probably Yes (PY) / Probably No (PN) / No (N) /No Information (NI)**   NA  NA  NA |
| **2.5 If Y/PY/NI to 2.4: Were these deviations from intended intervention balanced between groups?**  Pain scores  Supplemental analgesia  Adverse effects | **Not applicable (NA) / Yes (Y) / Probably Yes (PY) / Probably No (PN) / No (N) /No Information (NI)**  NA  NA  NA |
| **2.6 Was an appropriate analysis used to estimate the effect of assignment to intervention?**  Pain scores  Supplemental analgesia  Adverse effects | **Yes (Y) / Probably Yes (PY) / Probably No (PN) / No (N) / No Information (NI)**  Y  Y  Y |
| **2.7 If N/PN/NI to 2.6: Was there potential for a substantial impact (on the result) of the failure to analyse participants in the group to which they were randomized?**  Pain scores  Supplemental analgesia  Adverse effects | **Not applicable (NA) / Yes (Y) / Probably Yes (PY) / Probably No (PN) / No (N) /No Information (NI)**  NA  NA  NA |
| **Risk-of-bias judgement (domain 2)**  Pain scores  Supplemental analgesia  Adverse effects | **Low / High / Some concerns**  Low  Low  Low |
| **Risk of bias due to missing outcome data (domain 3)** |  |
| **3.1 Were data for this outcome available for all, or nearly all, participants randomized?**  Pain scores  Supplemental analgesia  Adverse effects | **Yes (Y) / Probably Yes (PY) / Probably No (PN) / No (N) / No Information (NI)**  Y  Y  Y |
| **3.2 If N/PN/NI to 3.1: Is there evidence that the result was not biased by missing outcome data?**  Pain scores  Supplemental analgesia  Adverse effects | **Not applicable (NA) / Yes (Y) / Probably Yes (PY) / Probably No (PN) / No (N) /No Information (NI)**  NA  NA  NA |
| **3.3 If N/PN to 3.2: Could missingness in the outcome depend on its true value?**  Pain scores  Supplemental analgesia  Adverse effects | **Not applicable (NA) / Yes (Y) / Probably Yes (PY) / Probably No (PN) / No (N) /No Information (NI)**   NA  NA  NA |
| **3.4 If Y/PY/NI to 3.3: Is it likely that missingness in the outcome depended on its true value?**  Pain scores  Supplemental analgesia  Adverse effects | **Not applicable (NA) / Yes (Y) / Probably Yes (PY) / Probably No (PN) / No (N) /No Information (NI)**  NA  NA  NA |
| **Risk-of-bias judgement (domain 3)**  Pain scores  Supplemental analgesia  Adverse effects | **Low / High / Some concerns**  Low  Low  Low |
| **Risk of bias in measurement of the outcome (domain 4)** | |
| **4.1 Was the method of measuring the outcome inappropriate?**  Pain scores  Supplemental analgesia  Adverse effects | **Yes (Y) / Probably Yes (PY) / Probably No (PN) / No (N) / No Information (NI)**  PN  N  N |
| **4.2 Could measurement or ascertainment of the outcome have differed between intervention groups?**  Pain scores  Supplemental analgesia  Adverse effects | **Yes (Y) / Probably Yes (PY) / Probably No (PN) / No (N) / No Information (NI)**  N  N  N |
| **4.3 If N/PN/NI to 4.1 or 4.2: Were outcome assessors aware of the intervention received by study participants?**  Pain scores  Supplemental analgesia  Adverse effects | **Not applicable (NA) / Yes (Y) / Probably Yes (PY) / Probably No (PN) / No (N) /No Information (NI)**  N  N  N |
| **4.4 If Y/PY/NI to 4.3: Could assessment of the outcome have been influenced by knowledge of intervention received?**  Pain scores  Supplemental analgesia  Adverse effects | **Not applicable (NA) / Yes (Y) / Probably Yes (PY) / Probably No (PN) / No (N) /No Information (NI)**  NA  NA  NA |
| **4.5 If Y/PY/NI to 4.4: Is it likely that assessment of the outcome was influenced by knowledge of intervention received?**  Pain scores  Supplemental analgesia  Adverse effects | **Not applicable (NA) / Yes (Y) / Probably Yes (PY) / Probably No (PN) / No (N) /No Information (NI)**  NA  NA  NA |
| **Risk-of-bias judgement (domain 4)**  Pain scores  Supplemental analgesia  Adverse effects | **Low / High / Some concerns**  Low  Low  Low |
| **Risk of bias in selection of the reported result (domain 5)** |  |
| **5.1 Were the data that produced this result analysed in accordance with a pre-specified analysis plan that was finalized before unblinded outcome data were available for analysis?**  Pain scores  Supplemental analgesia  Adverse effects | **Yes (Y) / Probably Yes (PY) / Probably No (PN) / No (N) / No Information (NI)**    NI  NI  NI |
| **5.2 Is the numerical result being assessed likely to have been selected, on the basis of the results, from multiple eligible outcome measurements (e.g. scales, definitions, time points) within the outcome domain?**  Pain scores  Supplemental analgesia  Adverse effects | **Yes (Y) / Probably Yes (PY) / Probably No (PN) / No (N) / No Information (NI)**    PN  N  N |
| **5.3 Is the numerical result being assessed likely to have been selected, on the basis of the results, from multiple eligible analyses of the data?**  Pain scores  Supplemental analgesia  Adverse effects | **Yes (Y) / Probably Yes (PY) / Probably No (PN) / No (N) / No Information (NI)**  PN  N  N |
| **Risk-of-bias judgement (domain 5)**  Pain scores  Supplemental analgesia  Adverse effects | **Low / High / Some concerns**  Some concerns  Some concerns  Some concerns |
| **Overall risk of bias based on the previous domains (domain 6)** |  |
| **Overall risk of bias**  Pain scores  Supplemental analgesia  Adverse effects | **Low / High / Some concerns**  Some concerns  Some concerns  Some concerns |

**Study: Liu, 2024^17^**

| **Risk of bias arising from the randomization process (domain 1)** | |
| --- | --- |
| **1.1 Was the allocation sequence random?**  General evaluation | **Yes (Y) / Probably Yes (PY) / Probably No (PN) / No (N) / No Information (NI)**  Y |
| **1.2 Was the allocation sequence concealed until participants were enrolled and assigned to interventions?**  General evaluation | **Yes (Y) / Probably Yes (PY) / Probably No (PN) / No (N) / No Information (NI)**   Y |
| **1.3 Did baseline differences between intervention groups suggest a problem with the randomization process?**  General evaluation | **Yes (Y) / Probably Yes (PY) / Probably No (PN) / No (N) / No Information (NI)**   N |
| **Risk-of-bias judgement (domain 1)**  Supplemental analgesia  Adverse effects | **Low / High / Some concerns**  Low  Low |
| **Risk of bias due to deviations from the intended interventions of assignment to intervention (domain 2)** | |
| **2.1 Were participants aware of their assigned intervention during the trial?**  General evaluation | **Yes (Y) / Probably Yes (PY) / Probably No (PN) / No (N) / No Information (NI)**  N |
| **2.2 Were carers and people delivering the interventions aware of participants’ assigned intervention during the trial?**  General evaluation | **Yes (Y) / Probably Yes (PY) / Probably No (PN) / No (N) / No Information (NI)**   N |
| **2.3 If Y/PY/NI to 2.1 or 2.2: Were there deviations from the intended intervention that arose because of the trial context?**  Supplemental analgesia  Adverse effects | **Not applicable (NA) / Yes (Y) / Probably Yes (PY) / Probably No (PN) / No (N) /No Information (NI)**  NA  NA |
| **2.4 If Y/PY to 2.3: Were these deviations likely to have affected the outcome?**  Supplemental analgesia  Adverse effects | **Not applicable (NA) / Yes (Y) / Probably Yes (PY) / Probably No (PN) / No (N) /No Information (NI)**  NA  NA |
| **2.5 If Y/PY/NI to 2.4: Were these deviations from intended intervention balanced between groups?**  Supplemental analgesia  Adverse effects | **Not applicable (NA) / Yes (Y) / Probably Yes (PY) / Probably No (PN) / No (N) /No Information (NI)**  NA  NA |
| **2.6 Was an appropriate analysis used to estimate the effect of assignment to intervention?**  Supplemental analgesia  Adverse effects | **Yes (Y) / Probably Yes (PY) / Probably No (PN) / No (N) / No Information (NI)**  Y  Y |
| **2.7 If N/PN/NI to 2.6: Was there potential for a substantial impact (on the result) of the failure to analyse participants in the group to which they were randomized?**  Supplemental analgesia  Adverse effects | **Not applicable (NA) / Yes (Y) / Probably Yes (PY) / Probably No (PN) / No (N) /No Information (NI)**  NA  NA |
| **Risk-of-bias judgement (domain 2)**  Supplemental analgesia  Adverse effects | **Low / High / Some concerns**  Low  Low |
| **Risk of bias due to missing outcome data (domain 3)** |  |
| **3.1 Were data for this outcome available for all, or nearly all, participants randomized?**  Supplemental analgesia  Adverse effects | **Yes (Y) / Probably Yes (PY) / Probably No (PN) / No (N) / No Information (NI)**  N  N |
| **3.2 If N/PN/NI to 3.1: Is there evidence that the result was not biased by missing outcome data?**  Supplemental analgesia  Adverse effects | **Not applicable (NA) / Yes (Y) / Probably Yes (PY) / Probably No (PN) / No (N) /No Information (NI)**  Y  Y |
| **3.3 If N/PN to 3.2: Could missingness in the outcome depend on its true value?**  Supplemental analgesia  Adverse effects | **Not applicable (NA) / Yes (Y) / Probably Yes (PY) / Probably No (PN) / No (N) /No Information (NI)**   NA  NA |
| **3.4 If Y/PY/NI to 3.3: Is it likely that missingness in the outcome depended on its true value?**  Supplemental analgesia  Adverse effects | **Not applicable (NA) / Yes (Y) / Probably Yes (PY) / Probably No (PN) / No (N) /No Information (NI)**  NA  NA |
| **Risk-of-bias judgement (domain 3)**  Supplemental analgesia  Adverse effects | **Low / High / Some concerns**  Low  Low |
| **Risk of bias in measurement of the outcome (domain 4)** | |
| **4.1 Was the method of measuring the outcome inappropriate?**  Supplemental analgesia  Adverse effects | **Yes (Y) / Probably Yes (PY) / Probably No (PN) / No (N) / No Information (NI)**  N  N |
| **4.2 Could measurement or ascertainment of the outcome have differed between intervention groups?**  Supplemental analgesia  Adverse effects | **Yes (Y) / Probably Yes (PY) / Probably No (PN) / No (N) / No Information (NI)**  N  N |
| **4.3 If N/PN/NI to 4.1 or 4.2: Were outcome assessors aware of the intervention received by study participants?**  Supplemental analgesia  Adverse effects | **Not applicable (NA) / Yes (Y) / Probably Yes (PY) / Probably No (PN) / No (N) /No Information (NI)**  N  PN |
| **4.4 If Y/PY/NI to 4.3: Could assessment of the outcome have been influenced by knowledge of intervention received?**  Supplemental analgesia  Adverse effects | **Not applicable (NA) / Yes (Y) / Probably Yes (PY) / Probably No (PN) / No (N) /No Information (NI)**  NA  NA |
| **4.5 If Y/PY/NI to 4.4: Is it likely that assessment of the outcome was influenced by knowledge of intervention received?**  Supplemental analgesia  Adverse effects | **Not applicable (NA) / Yes (Y) / Probably Yes (PY) / Probably No (PN) / No (N) /No Information (NI)**  NA  NA |
| **Risk-of-bias judgement (domain 4)**  Supplemental analgesia  Adverse effects | **Low / High / Some concerns**  Low  Low |
| **Risk of bias in selection of the reported result (domain 5)** |  |
| **5.1 Were the data that produced this result analysed in accordance with a pre-specified analysis plan that was finalized before unblinded outcome data were available for analysis?**  Supplemental analgesia  Adverse effects | **Yes (Y) / Probably Yes (PY) / Probably No (PN) / No (N) / No Information (NI)**    Y  Y |
| **5.2 Is the numerical result being assessed likely to have been selected, on the basis of the results, from multiple eligible outcome measurements (e.g. scales, definitions, time points) within the outcome domain?**  Supplemental analgesia  Adverse effects | **Yes (Y) / Probably Yes (PY) / Probably No (PN) / No (N) / No Information (NI)**    N  N |
| **5.3 Is the numerical result being assessed likely to have been selected, on the basis of the results, from multiple eligible analyses of the data?**  Supplemental analgesia  Adverse effects | **Yes (Y) / Probably Yes (PY) / Probably No (PN) / No (N) / No Information (NI)**  N  N |
| **Risk-of-bias judgement (domain 5)**  Supplemental analgesia  Adverse effects | **Low / High / Some concerns**  Low  Low |
| **Overall risk of bias based on the previous domains (domain 6)** |  |
| **Overall risk of bias**  Supplemental analgesia  Adverse effects | **Low / High / Some concerns**  Low  Low |

**Study: Marinangeli, 2002^18^**

| **Risk of bias arising from the randomization process (domain 1)** | |
| --- | --- |
| **1.1 Was the allocation sequence random?**  General evaluation | **Yes (Y) / Probably Yes (PY) / Probably No (PN) / No (N) / No Information (NI)**  NI |
| **1.2 Was the allocation sequence concealed until participants were enrolled and assigned to interventions?**  General evaluation | **Yes (Y) / Probably Yes (PY) / Probably No (PN) / No (N) / No Information (NI)**   NI |
| **1.3 Did baseline differences between intervention groups suggest a problem with the randomization process?**  General evaluation | **Yes (Y) / Probably Yes (PY) / Probably No (PN) / No (N) / No Information (NI)**   N |
| **Risk-of-bias judgement (domain 1)**  Pain scores  Supplemental analgesia  Adverse effects | **Low / High / Some concerns**  Some concerns  Some concerns  Some concerns |
| **Risk of bias due to deviations from the intended interventions of assignment to intervention (domain 2)** | |
| **2.1 Were participants aware of their assigned intervention during the trial?**  General evaluation | **Yes (Y) / Probably Yes (PY) / Probably No (PN) / No (N) / No Information (NI)**  N |
| **2.2 Were carers and people delivering the interventions aware of participants’ assigned intervention during the trial?**  General evaluation | **Yes (Y) / Probably Yes (PY) / Probably No (PN) / No (N) / No Information (NI)**   N |
| **2.3 If Y/PY/NI to 2.1 or 2.2: Were there deviations from the intended intervention that arose because of the trial context?**  Pain scores  Supplemental analgesia  Adverse effects | **Not applicable (NA) / Yes (Y) / Probably Yes (PY) / Probably No (PN) / No (N) /No Information (NI)**  NA  NA  NA |
| **2.4 If Y/PY to 2.3: Were these deviations likely to have affected the outcome?**  Pain scores  Supplemental analgesia  Adverse effects | **Not applicable (NA) / Yes (Y) / Probably Yes (PY) / Probably No (PN) / No (N) /No Information (NI)**   NA  NA  NA |
| **2.5 If Y/PY/NI to 2.4: Were these deviations from intended intervention balanced between groups?**  Pain scores  Supplemental analgesia  Adverse effects | **Not applicable (NA) / Yes (Y) / Probably Yes (PY) / Probably No (PN) / No (N) /No Information (NI)**  NA  NA  NA |
| **2.6 Was an appropriate analysis used to estimate the effect of assignment to intervention?**  Pain scores  Supplemental analgesia  Adverse effects | **Yes (Y) / Probably Yes (PY) / Probably No (PN) / No (N) / No Information (NI)**  PY  PY  PY |
| **2.7 If N/PN/NI to 2.6: Was there potential for a substantial impact (on the result) of the failure to analyse participants in the group to which they were randomized?**  Pain scores  Supplemental analgesia  Adverse effects | **Not applicable (NA) / Yes (Y) / Probably Yes (PY) / Probably No (PN) / No (N) /No Information (NI)**  NA  NA  NA |
| **Risk-of-bias judgement (domain 2)**  Pain scores  Supplemental analgesia  Adverse effects | **Low / High / Some concerns**  Low  Low  Low |
| **Risk of bias due to missing outcome data (domain 3)** |  |
| **3.1 Were data for this outcome available for all, or nearly all, participants randomized?**  Pain scores  Supplemental analgesia  Adverse effects | **Yes (Y) / Probably Yes (PY) / Probably No (PN) / No (N) / No Information (NI)**  Y  Y  PY |
| **3.2 If N/PN/NI to 3.1: Is there evidence that the result was not biased by missing outcome data?**  Pain scores  Supplemental analgesia  Adverse effects | **Not applicable (NA) / Yes (Y) / Probably Yes (PY) / Probably No (PN) / No (N) /No Information (NI)**  NA  NA  NA |
| **3.3 If N/PN to 3.2: Could missingness in the outcome depend on its true value?**  Pain scores  Supplemental analgesia  Adverse effects | **Not applicable (NA) / Yes (Y) / Probably Yes (PY) / Probably No (PN) / No (N) /No Information (NI)**   NA  NA  NA |
| **3.4 If Y/PY/NI to 3.3: Is it likely that missingness in the outcome depended on its true value?**  Pain scores  Supplemental analgesia  Adverse effects | **Not applicable (NA) / Yes (Y) / Probably Yes (PY) / Probably No (PN) / No (N) /No Information (NI)**  NA  NA  NA |
| **Risk-of-bias judgement (domain 3)**  Pain scores  Supplemental analgesia  Adverse effects | **Low / High / Some concerns**  Low  Low  Low |
| **Risk of bias in measurement of the outcome (domain 4)** | |
| **4.1 Was the method of measuring the outcome inappropriate?**  Pain scores  Supplemental analgesia  Adverse effects | **Yes (Y) / Probably Yes (PY) / Probably No (PN) / No (N) / No Information (NI)**  N  N  PN |
| **4.2 Could measurement or ascertainment of the outcome have differed between intervention groups?**  Pain scores  Supplemental analgesia  Adverse effects | **Yes (Y) / Probably Yes (PY) / Probably No (PN) / No (N) / No Information (NI)**  N  N  N |
| **4.3 If N/PN/NI to 4.1 or 4.2: Were outcome assessors aware of the intervention received by study participants?**  Pain scores  Supplemental analgesia  Adverse effects | **Not applicable (NA) / Yes (Y) / Probably Yes (PY) / Probably No (PN) / No (N) /No Information (NI)**  N  N  N |
| **4.4 If Y/PY/NI to 4.3: Could assessment of the outcome have been influenced by knowledge of intervention received?**  Pain scores  Supplemental analgesia  Adverse effects | **Not applicable (NA) / Yes (Y) / Probably Yes (PY) / Probably No (PN) / No (N) /No Information (NI)**  NA  NA  NA |
| **4.5 If Y/PY/NI to 4.4: Is it likely that assessment of the outcome was influenced by knowledge of intervention received?**  Pain scores  Supplemental analgesia  Adverse effects | **Not applicable (NA) / Yes (Y) / Probably Yes (PY) / Probably No (PN) / No (N) /No Information (NI)**  NA  NA  NA |
| **Risk-of-bias judgement (domain 4)**  Pain scores  Supplemental analgesia  Adverse effects | **Low / High / Some concerns**  Low  Low  Low |
| **Risk of bias in selection of the reported result (domain 5)** |  |
| **5.1 Were the data that produced this result analysed in accordance with a pre-specified analysis plan that was finalized before unblinded outcome data were available for analysis?**  Pain scores  Supplemental analgesia  Adverse effects | **Yes (Y) / Probably Yes (PY) / Probably No (PN) / No (N) / No Information (NI)**    NI  NI  NI |
| **5.2 Is the numerical result being assessed likely to have been selected, on the basis of the results, from multiple eligible outcome measurements (e.g. scales, definitions, time points) within the outcome domain?**  Pain scores  Supplemental analgesia  Adverse effects | **Yes (Y) / Probably Yes (PY) / Probably No (PN) / No (N) / No Information (NI)**    PN  PN  PN |
| **5.3 Is the numerical result being assessed likely to have been selected, on the basis of the results, from multiple eligible analyses of the data?**  Pain scores  Supplemental analgesia  Adverse effects | **Yes (Y) / Probably Yes (PY) / Probably No (PN) / No (N) / No Information (NI)**  PN  PN  N |
| **Risk-of-bias judgement (domain 5)**  Pain scores  Supplemental analgesia  Adverse effects | **Low / High / Some concerns**  Some concerns  Some concerns  Some concerns |
| **Overall risk of bias based on the previous domains (domain 6)** |  |
| **Overall risk of bias**  Pain scores  Supplemental analgesia  Adverse effects | **Low / High / Some concerns**  Some concerns  Some concerns  Some concerns |

**Study: Salengros, 2011^24^**

| **Risk of bias arising from the randomization process (domain 1)** | |
| --- | --- |
| **1.1 Was the allocation sequence random?**  General evaluation | **Yes (Y) / Probably Yes (PY) / Probably No (PN) / No (N) / No Information (NI)**  Y |
| **1.2 Was the allocation sequence concealed until participants were enrolled and assigned to interventions?**  General evaluation | **Yes (Y) / Probably Yes (PY) / Probably No (PN) / No (N) / No Information (NI)**   Y |
| **1.3 Did baseline differences between intervention groups suggest a problem with the randomization process?**  General evaluation | **Yes (Y) / Probably Yes (PY) / Probably No (PN) / No (N) / No Information (NI)**   N |
| **Risk-of-bias judgement (domain 1)**  Pain scores  Supplemental analgesia  Adverse effects | **Low / High / Some concerns**  Low  Low  Low |
| **Risk of bias due to deviations from the intended interventions of assignment to intervention (domain 2)** | |
| **2.1 Were participants aware of their assigned intervention during the trial?**  General evaluation | **Yes (Y) / Probably Yes (PY) / Probably No (PN) / No (N) / No Information (NI)**  N |
| **2.2 Were carers and people delivering the interventions aware of participants’ assigned intervention during the trial?**  General evaluation | **Yes (Y) / Probably Yes (PY) / Probably No (PN) / No (N) / No Information (NI)**   N |
| **2.3 If Y/PY/NI to 2.1 or 2.2: Were there deviations from the intended intervention that arose because of the trial context?**  Pain scores  Supplemental analgesia  Adverse effects | **Not applicable (NA) / Yes (Y) / Probably Yes (PY) / Probably No (PN) / No (N) /No Information (NI)**  NA  NA  NA |
| **2.4 If Y/PY to 2.3: Were these deviations likely to have affected the outcome?**  Pain scores  Supplemental analgesia  Adverse effects | **Not applicable (NA) / Yes (Y) / Probably Yes (PY) / Probably No (PN) / No (N) /No Information (NI)**   NA  NA  NA |
| **2.5 If Y/PY/NI to 2.4: Were these deviations from intended intervention balanced between groups?**  Pain scores  Supplemental analgesia  Adverse effects | **Not applicable (NA) / Yes (Y) / Probably Yes (PY) / Probably No (PN) / No (N) /No Information (NI)**  NA  NA  NA |
| **2.6 Was an appropriate analysis used to estimate the effect of assignment to intervention?**  Pain scores  Supplemental analgesia  Adverse effects | **Yes (Y) / Probably Yes (PY) / Probably No (PN) / No (N) / No Information (NI)**  Y  Y  Y |
| **2.7 If N/PN/NI to 2.6: Was there potential for a substantial impact (on the result) of the failure to analyse participants in the group to which they were randomized?**  Pain scores  Supplemental analgesia  Adverse effects | **Not applicable (NA) / Yes (Y) / Probably Yes (PY) / Probably No (PN) / No (N) /No Information (NI)**  NA  NA  NA |
| **Risk-of-bias judgement (domain 2)**  Pain scores  Supplemental analgesia  Adverse effects | **Low / High / Some concerns**  Low  Low  Low |
| **Risk of bias due to missing outcome data (domain 3)** |  |
| **3.1 Were data for this outcome available for all, or nearly all, participants randomized?**  Pain scores  Supplemental analgesia  Adverse effects | **Yes (Y) / Probably Yes (PY) / Probably No (PN) / No (N) / No Information (NI)**  Y  Y  Y |
| **3.2 If N/PN/NI to 3.1: Is there evidence that the result was not biased by missing outcome data?**  Pain scores  Supplemental analgesia  Adverse effects | **Not applicable (NA) / Yes (Y) / Probably Yes (PY) / Probably No (PN) / No (N) /No Information (NI)**  NA  NA  NA |
| **3.3 If N/PN to 3.2: Could missingness in the outcome depend on its true value?**  Pain scores  Supplemental analgesia  Adverse effects | **Not applicable (NA) / Yes (Y) / Probably Yes (PY) / Probably No (PN) / No (N) /No Information (NI)**   NA  NA  NA |
| **3.4 If Y/PY/NI to 3.3: Is it likely that missingness in the outcome depended on its true value?**  Pain scores  Supplemental analgesia  Adverse effects | **Not applicable (NA) / Yes (Y) / Probably Yes (PY) / Probably No (PN) / No (N) /No Information (NI)**  NA  NA  NA |
| **Risk-of-bias judgement (domain 3)**  Pain scores  Supplemental analgesia  Adverse effects | **Low / High / Some concerns**  Low  Low  Low |
| **Risk of bias in measurement of the outcome (domain 4)** | |
| **4.1 Was the method of measuring the outcome inappropriate?**  Pain scores  Supplemental analgesia  Adverse effects | **Yes (Y) / Probably Yes (PY) / Probably No (PN) / No (N) / No Information (NI)**  N  N  N |
| **4.2 Could measurement or ascertainment of the outcome have differed between intervention groups?**  Pain scores  Supplemental analgesia  Adverse effects | **Yes (Y) / Probably Yes (PY) / Probably No (PN) / No (N) / No Information (NI)**  N  N  N |
| **4.3 If N/PN/NI to 4.1 or 4.2: Were outcome assessors aware of the intervention received by study participants?**  Pain scores  Supplemental analgesia  Adverse effects | **Not applicable (NA) / Yes (Y) / Probably Yes (PY) / Probably No (PN) / No (N) /No Information (NI)**  N  N  N |
| **4.4 If Y/PY/NI to 4.3: Could assessment of the outcome have been influenced by knowledge of intervention received?**  Pain scores  Supplemental analgesia  Adverse effects | **Not applicable (NA) / Yes (Y) / Probably Yes (PY) / Probably No (PN) / No (N) /No Information (NI)**  NA  NA  NA |
| **4.5 If Y/PY/NI to 4.4: Is it likely that assessment of the outcome was influenced by knowledge of intervention received?**  Pain scores  Supplemental analgesia  Adverse effects | **Not applicable (NA) / Yes (Y) / Probably Yes (PY) / Probably No (PN) / No (N) /No Information (NI)**  NA  NA  NA |
| **Risk-of-bias judgement (domain 4)**  Pain scores  Supplemental analgesia  Adverse effects | **Low / High / Some concerns**  Low  Low  Low |
| **Risk of bias in selection of the reported result (domain 5)** |  |
| **5.1 Were the data that produced this result analysed in accordance with a pre-specified analysis plan that was finalized before unblinded outcome data were available for analysis?**  Pain scores  Supplemental analgesia  Adverse effects | **Yes (Y) / Probably Yes (PY) / Probably No (PN) / No (N) / No Information (NI)**    Y  PY  NI |
| **5.2 Is the numerical result being assessed likely to have been selected, on the basis of the results, from multiple eligible outcome measurements (e.g. scales, definitions, time points) within the outcome domain?**  Pain scores  Supplemental analgesia  Adverse effects | **Yes (Y) / Probably Yes (PY) / Probably No (PN) / No (N) / No Information (NI)**    N  N  PN |
| **5.3 Is the numerical result being assessed likely to have been selected, on the basis of the results, from multiple eligible analyses of the data?**  Pain scores  Supplemental analgesia  Adverse effects | **Yes (Y) / Probably Yes (PY) / Probably No (PN) / No (N) / No Information (NI)**  PN  PN  PN |
| **Risk-of-bias judgement (domain 5)**  Pain scores  Supplemental analgesia  Adverse effects | **Low / High / Some concerns**  Low  Low  Some concerns |
| **Overall risk of bias based on the previous domains (domain 6)** |  |
| **Overall risk of bias**  Pain scores  Supplemental analgesia  Adverse effects | **Low / High / Some concerns**  Low  Low  Some concerns |

**Study: Striebel, 1993^19^**

| **Risk of bias arising from the randomization process (domain 1)** | |
| --- | --- |
| **1.1 Was the allocation sequence random?**  General evaluation | **Yes (Y) / Probably Yes (PY) / Probably No (PN) / No (N) / No Information (NI)**  NI |
| **1.2 Was the allocation sequence concealed until participants were enrolled and assigned to interventions?**  General evaluation | **Yes (Y) / Probably Yes (PY) / Probably No (PN) / No (N) / No Information (NI)**   NI |
| **1.3 Did baseline differences between intervention groups suggest a problem with the randomization process?**  General evaluation | **Yes (Y) / Probably Yes (PY) / Probably No (PN) / No (N) / No Information (NI)**   N |
| **Risk-of-bias judgement (domain 1)**  Pain scores  Supplemental analgesia  Adverse effects | **Low / High / Some concerns**  Some concerns  Some concerns  Some concerns |
| **Risk of bias due to deviations from the intended interventions of assignment to intervention (domain 2)** | |
| **2.1 Were participants aware of their assigned intervention during the trial?**  General evaluation | **Yes (Y) / Probably Yes (PY) / Probably No (PN) / No (N) / No Information (NI)**  PN |
| **2.2 Were carers and people delivering the interventions aware of participants’ assigned intervention during the trial?**  General evaluation | **Yes (Y) / Probably Yes (PY) / Probably No (PN) / No (N) / No Information (NI)**   PN |
| **2.3 If Y/PY/NI to 2.1 or 2.2: Were there deviations from the intended intervention that arose because of the trial context?**  Pain scores  Supplemental analgesia  Adverse effects | **Not applicable (NA) / Yes (Y) / Probably Yes (PY) / Probably No (PN) / No (N) /No Information (NI)**  NA  NA  NA |
| **2.4 If Y/PY to 2.3: Were these deviations likely to have affected the outcome?**  Pain scores  Supplemental analgesia  Adverse effects | **Not applicable (NA) / Yes (Y) / Probably Yes (PY) / Probably No (PN) / No (N) /No Information (NI)**   NA  NA  NA |
| **2.5 If Y/PY/NI to 2.4: Were these deviations from intended intervention balanced between groups?**  Pain scores  Supplemental analgesia  Adverse effects | **Not applicable (NA) / Yes (Y) / Probably Yes (PY) / Probably No (PN) / No (N) /No Information (NI)**  NA  NA  NA |
| **2.6 Was an appropriate analysis used to estimate the effect of assignment to intervention?**  Pain scores  Supplemental analgesia  Adverse effects | **Yes (Y) / Probably Yes (PY) / Probably No (PN) / No (N) / No Information (NI)**  Y  Y  PY |
| **2.7 If N/PN/NI to 2.6: Was there potential for a substantial impact (on the result) of the failure to analyse participants in the group to which they were randomized?**  Pain scores  Supplemental analgesia  Adverse effects | **Not applicable (NA) / Yes (Y) / Probably Yes (PY) / Probably No (PN) / No (N) /No Information (NI)**  NA  NA  NA |
| **Risk-of-bias judgement (domain 2)**  Pain scores  Supplemental analgesia  Adverse effects | **Low / High / Some concerns**  Low  Low  Low |
| **Risk of bias due to missing outcome data (domain 3)** |  |
| **3.1 Were data for this outcome available for all, or nearly all, participants randomized?**  Pain scores  Supplemental analgesia  Adverse effects | **Yes (Y) / Probably Yes (PY) / Probably No (PN) / No (N) / No Information (NI)**  PY  Y  Y |
| **3.2 If N/PN/NI to 3.1: Is there evidence that the result was not biased by missing outcome data?**  Pain scores  Supplemental analgesia  Adverse effects | **Not applicable (NA) / Yes (Y) / Probably Yes (PY) / Probably No (PN) / No (N) /No Information (NI)**  NA  NA  NA |
| **3.3 If N/PN to 3.2: Could missingness in the outcome depend on its true value?**  Pain scores  Supplemental analgesia  Adverse effects | **Not applicable (NA) / Yes (Y) / Probably Yes (PY) / Probably No (PN) / No (N) /No Information (NI)**   NA  NA  NA |
| **3.4 If Y/PY/NI to 3.3: Is it likely that missingness in the outcome depended on its true value?**  Pain scores  Supplemental analgesia  Adverse effects | **Not applicable (NA) / Yes (Y) / Probably Yes (PY) / Probably No (PN) / No (N) /No Information (NI)**  NA  NA  NA |
| **Risk-of-bias judgement (domain 3)**  Pain scores  Supplemental analgesia  Adverse effects | **Low / High / Some concerns**  Low  Low  Low |
| **Risk of bias in measurement of the outcome (domain 4)** | |
| **4.1 Was the method of measuring the outcome inappropriate?**  Pain scores  Supplemental analgesia  Adverse effects | **Yes (Y) / Probably Yes (PY) / Probably No (PN) / No (N) / No Information (NI)**  N  N  PN |
| **4.2 Could measurement or ascertainment of the outcome have differed between intervention groups?**  Pain scores  Supplemental analgesia  Adverse effects | **Yes (Y) / Probably Yes (PY) / Probably No (PN) / No (N) / No Information (NI)**  N  N  PN |
| **4.3 If N/PN/NI to 4.1 or 4.2: Were outcome assessors aware of the intervention received by study participants?**  Pain scores  Supplemental analgesia  Adverse effects | **Not applicable (NA) / Yes (Y) / Probably Yes (PY) / Probably No (PN) / No (N) /No Information (NI)**  N  N  N |
| **4.4 If Y/PY/NI to 4.3: Could assessment of the outcome have been influenced by knowledge of intervention received?**  Pain scores  Supplemental analgesia  Adverse effects | **Not applicable (NA) / Yes (Y) / Probably Yes (PY) / Probably No (PN) / No (N) /No Information (NI)**  NA  NA  NA |
| **4.5 If Y/PY/NI to 4.4: Is it likely that assessment of the outcome was influenced by knowledge of intervention received?**  Pain scores  Supplemental analgesia  Adverse effects | **Not applicable (NA) / Yes (Y) / Probably Yes (PY) / Probably No (PN) / No (N) /No Information (NI)**  NA  NA  NA |
| **Risk-of-bias judgement (domain 4)**  Pain scores  Supplemental analgesia  Adverse effects | **Low / High / Some concerns**  Low  Low  Low |
| **Risk of bias in selection of the reported result (domain 5)** |  |
| **5.1 Were the data that produced this result analysed in accordance with a pre-specified analysis plan that was finalized before unblinded outcome data were available for analysis?**  Pain scores  Supplemental analgesia  Adverse effects | **Yes (Y) / Probably Yes (PY) / Probably No (PN) / No (N) / No Information (NI)**    NI  NI  NI |
| **5.2 Is the numerical result being assessed likely to have been selected, on the basis of the results, from multiple eligible outcome measurements (e.g. scales, definitions, time points) within the outcome domain?**  Pain scores  Supplemental analgesia  Adverse effects | **Yes (Y) / Probably Yes (PY) / Probably No (PN) / No (N) / No Information (NI)**    PN  PN  PN |
| **5.3 Is the numerical result being assessed likely to have been selected, on the basis of the results, from multiple eligible analyses of the data?**  Pain scores  Supplemental analgesia  Adverse effects | **Yes (Y) / Probably Yes (PY) / Probably No (PN) / No (N) / No Information (NI)**  PN  PN  PN |
| **Risk-of-bias judgement (domain 5)**  Pain scores  Supplemental analgesia  Adverse effects | **Low / High / Some concerns**  Some concerns  Some concerns  Some concerns |
| **Overall risk of bias based on the previous domains (domain 6)** |  |
| **Overall risk of bias**  Pain scores  Supplemental analgesia  Adverse effects | **Low / High / Some concerns**  Some concerns  Some concerns  Some concerns |
